# Algebraic Representation of Mitochondrial Dynamics

**DOI:** 10.1007/s11538-026-01738-9

**Published:** 2026-09-04

**Authors:** Raphael Mostov, Greyson Lewis, Gabriel Sturm, Wallace F. Marshall

**Affiliations:** Department Biochemistry and Biophysics, University of California, San Francisco, USA

**Keywords:** Mitochondrial fission, Mitochondrial fusion, Budding yeast, Spatial statistics, Planar graphs, Cell representation, Algebraic graph theory, Morpholomics

## Abstract

This paper addresses the increasing need for comprehensive mathematical descriptions of cell organization by examining the algebraic structure of mitochondrial network dynamics. Mitochondria are cellular structures involved in metabolism that take the form of a network of membrane-based tubes that undergo continuous re-arrangement by a set of morphological processes, including fission and fusion, carried out by protein-based machinery. Because of their network structure, mitochondria can be represented as graphs, and the morphological operations that take place in the cell, referred to as mitochondrial dynamics, can be represented by changes to the graphs. Prior studies have classified mitochondrial graphs based on graph-theoretic features, but an alternative approach is to focus not on the graphs themselves but on the set of morphological operations inducing mitochondrial dynamics, since this may provide a simpler representation. Moreover, the operations are what determine the graphs that will be generated in a biological system. Here we show that mitochondrial dynamics give rise to a category in which the objects are equivalence classes of graphs defined by one of the morphological operations and morphisms are mappings between these equivalence classes defined by the remaining morphological operations. For mitochondria consisting of a single component this gives rise to a particularly simple representation. Using these formalisms we define a distance metric for similarity between mitochondrial structures based on an edit distance, and demonstrate how this representation can be used for visualization and statistical analysis of biological data. In the course of defining these structures we provide a mathematical motivation for new experimental questions regarding mitochondrial fusion, the impacts of cell division on mitochondrial morphology, and the presence of a single giant component in some cell types. This work points to a general strategy for formulating a cell structure state-space, based not on the shapes of cellular structures, but on relations between the dynamic operations that produce them.

## Introduction

### The Problem of Cell Representation

Cell biology is drowning in data. With the advancement of high-throughput imaging and super-resolution methods, we are reaching a point where the quantity of information generated becomes difficult to comprehend. There is growing interest in developing mathematical approaches for representing cell structure in simpler terms that can be more tractable (Viana et al. 2023; Rafelski and Theriot 2024; Johnson et al. 2015). A formal way to represent cell structure would enable using the representation to classify cell types or cell states; to quantify similarity and differences between cell organization under different conditions; and to provide a way to describe the complex phenotypic effects of mutations.

Formal cell representations also provide a basis for mathematical models that can help us to understand, predict, and engineer cell behavior. The type of mathematical model used for analyzing cell behavior depends on the underlying representation used for cell state. The vast majority of mathematical models in biology have relied on representing biological processes using continuous variables and systems of differential equations (Murray 2002). Many biological questions remain difficult to model in terms of differential equations, suggesting a need for alternative mathematical modeling frameworks better suited to such questions. One alternative type of mathematical approach is algebra, in which a biological system is represented in terms of groups or other algebraic structures (Danckwerts and Neubert 1975; Rietman et al. 2011; Reidun Twarock and Tom Keef 2007). Here we develop an approach to use abstract algebra to represent the dynamics of a key cellular structure - the mitochondrion.

### The Mitochondrion - An Organelle with a Network Morphology

Mitochondria, often referred to as the “powerhouse of the cell," are often depicted as bean-shaped objects in textbooks. But in reality, mitochondria in most cells exist as networks of tubules spread throughout the cell interior (Friedman and Nunnari 2014). Mitochondrial network morphology is conspicuously different when cells are grown under different conditions (Mishra and Chan 2014), and also changes during the cell cycle (Daciana et al. 2002), cell differentiation (Shin et al. 2016), and in various disease states (Peter et al. 2009). The connectivity of mitochondria in a cell determines the ability of metabolic products to move within them (Friedman and Nunnari 2014; Brown et al. 2020), as well as the ability of mitochondrial DNA to redistribute (Lieber et al. 2019), and to be inherited (Westermann 2010; Kornick et al. 2019). For all of these reasons, mitochondrial network morphology is biologically important, and the molecular mechanisms that produce these networks have been the topic of intense study.

What determines the network morphology of mitochondria (Lewis and Marshall 2023)? Like any physical network, we can describe mitochondrial morphology using the mathematical definition of a graph with vertices and edges (Viana et al. 2020; Rafelski et al. 2012). For mitochondria, these graphs consist entirely of vertices of degree 1 or 3 (Lewis and Marshall 2023). Vertices of degree 4 (four-way junctions) sometimes occur transiently but rapidly resolve into pairs of degree 3 vertices. In some organisms, the network is constrained to lie on the surface of the cell, such that the graph is planar.

#### Definition 1.1

A *mitochondria graph* is a graph composed exclusively of degree-one and degree-three vertices. The set of all such graphs we name *MG*

**Fig. 1:**
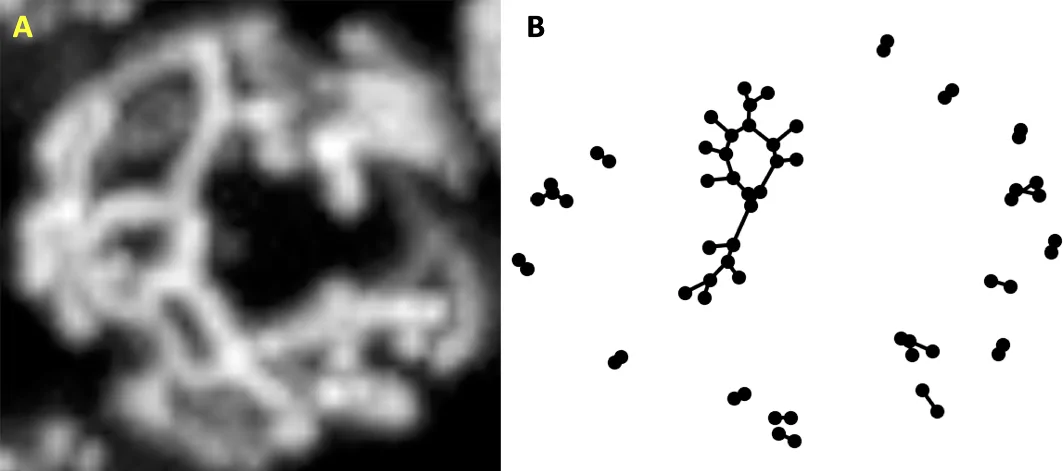
Graph representation of a mitochondria network. **A** max-intensity projection of 3D image of budding yeast cell expressing mito-dsRed, grown in glycerol and imaged by spinning disk confocal microscopy. **B** Mitochondrial graph structure of yeast cell in glycerol computed using MitoGraph 2 software (Viana et al. 2020)

Several software packages, including MitoGraph (Viana et al. 2020) and Lefebvre et al. (2021), can be used to convert 3D images of mitochondria into graph representations (See Fig. 1). This ability to render mitochondria as graphs makes it easy, in principle, to directly apply mathematical ideas from graph theory to actual biological data.

### Mitochondrial Dynamics

**Fig. 2:**
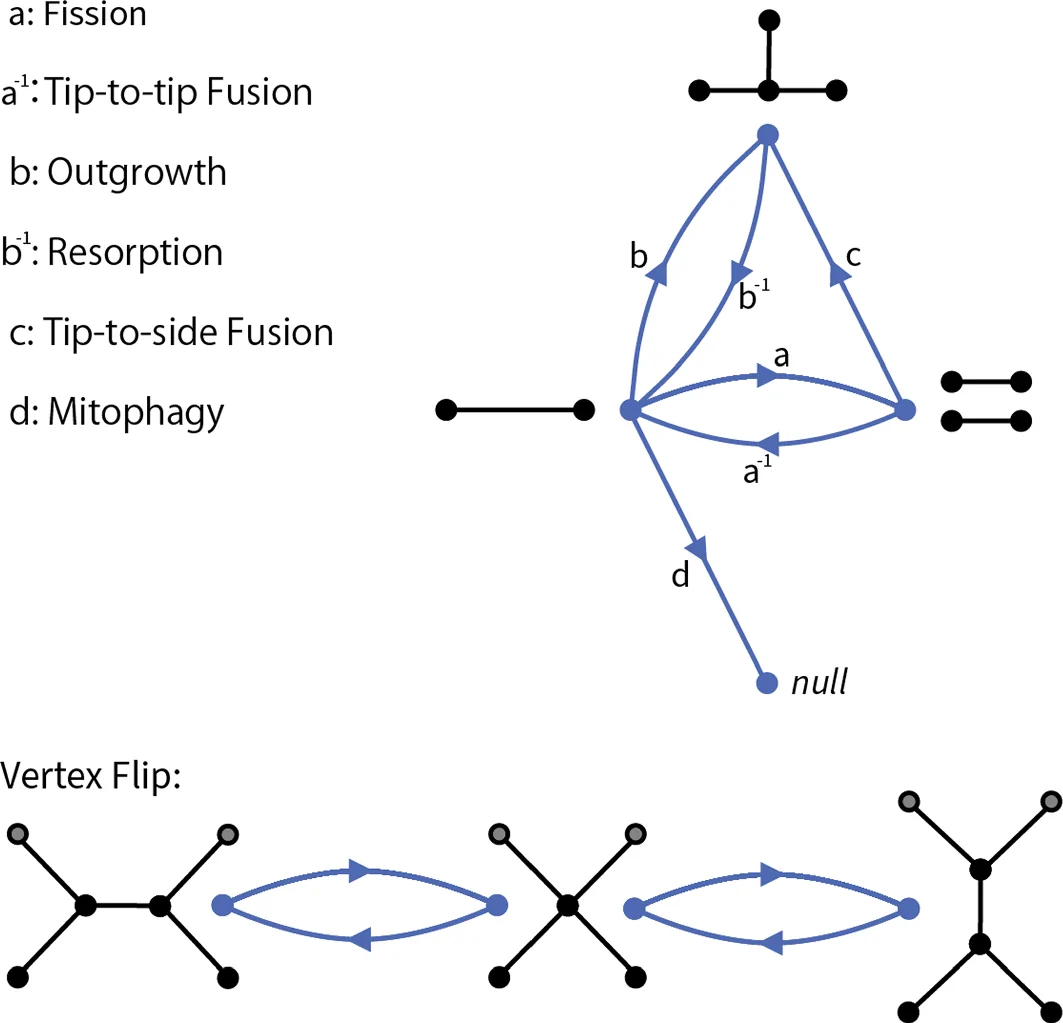
Morphological operations on mitochondria graphs (Color figure online)

One interesting feature of mitochondrial morphology is that the networks are dynamic, constantly changing their connectivity (Nunnari et al. 1997). This occurs primarily through fission, the splitting of network branches, and fusion, in which two network branches join together (Shaw and Nunnari 2002). There are two types of fusion: tip-to-tip, in which the ends of two different network branches join, and tip-to-side, where the end of one branch joins to the side of another. Additionally, there are several other morphological transformations; outgrowth: where a new branch grows out from the side of an existing branch; resorption: the inverse of outgrowth; mitophagy: the digestion and removal of small mitochondria; and the vertex flip, in which two vertices slide past each other due to branch migration, as diagrammed in Fig. 2. As shown in Fig. 2, we can describe these morphological transformations using the graph representation of mitochondrial networks (Lewis and Marshall 2023). We will use symbols to describe the morphological operations, such that a represents fission, b represents branch outgrowth, c represents tip-side fusion, d represents mitophagy, v represents vertex flip, and e represents the null operation in which the graph is left unchanged. Experimental studies show that altering the rates of fission and fusion processes gives rise to mitochondrial networks with different graph structures (Viana et al. 2020). Here we focus on this specific set of morphological operations, but mitochondrial dynamics remains a field of current investigation and we cannot rule out the possibility of other morphological operations not included in this list.

**Fig. 3:**
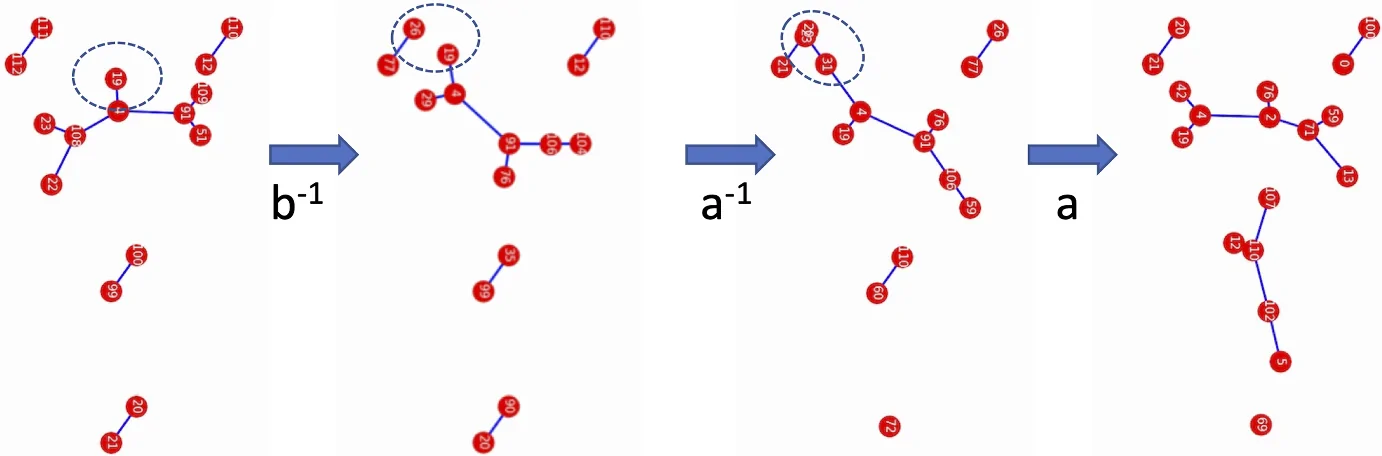
Graph dynamics in mitochondria in living cells. Images show four sequential 3D images of the mitochondrial network in a budding yeast cell. Strain: CGY49.77 wildtype with preCox4-mNeon-green & TIM20-mCherry, grown to stationary phase in synthetic complete medium supplemented with 2% glucose and 2% methyl-alpha-D-mannopyranoside, imaged by single objective light sheet microscopy at a rate of 1 3D volume per second, and graphs extracted using the Nellie software package (Lefebvre et al. 2021). Dotted circles indicate the region on each graph that is involved in the next operation as indicated by the letters below (Color figure online)

By acquiring 3D images of mitochondria in live cells over time, and then converting the mitochondrial network to a graph at each time-point, it is possible to represent mitochondrial dynamics as a series of the above-listed morphological operations. Figure 3 shows one example from an actual cell in which a branch resorption event is followed by fusion of the large component with a smaller component, which is then followed by fission. Technology to acquire 3D images of mitochondrial networks in living cells at high spatial and temporal resolution, and to convert these images into graph representations, is by now quite well developed. The challenge now is how to gain insights from such rapidly expanding datasets.

Consideration of mitochondria as dynamically changing graphs raises a number of questions that are inherently mathematical in nature. What is the range of possible mitochondrial graphs, and what statistical distribution of graphs is expected as a function of the rates of the dynamic processes? How do the different processes contribute to the space of possible graphs? Is there any sort of control system in the cell that actively adjusts the choice of operations to tune the network structure, or are the various morphological operations acting independently and at random?

Other questions are primarily biological: how can we compare different mitochondrial network morphologies seen in different growth conditions or disease states? How can we define the phenotype of mutations that affect mitochondrial network morphology and decide when two mutations have a similar effect and therefore may be acting in the same pathway? Are the types of networks that we see under some set of conditions consistent with some mechanistic model of how and when the different morphological transitions take place?

To answer these questions, it is necessary to understand the structure of the space of mitochondrial graphs and their transitions. However, the space of mitochondrial graphs is complicated, difficult to enumerate due to the problem of graph isomorphism, and, from a theoretical standpoint, infinite. First of all, the space of possible graphs becomes extremely large as the size of the graphs increases, even given the constraints on the degrees present in mitochondrial graphs. For example, the number of distinct graphs with 20 vertices is approximately $10^{34}$ (see Online Encylopedia of Integer Sequences). By comparison, the total number of cells, including bacteria and eukaryotes, is estimated to be roughly $10^{30}$ (Yinon et al. 2018). Even with restrictions imposed by the structures of mitographs, the number of possible graphs is still astronomically large, and this has a direct practical impact on biological research, because it means that in order to have high chance of observing the same graph more than once, one would need to image an impractically large number of cells. This means that the space of possible graphs will be sampled sparsely, creating huge difficulties in statistical comparison of the distribution of graphs between different strains or growth conditions.

**Fig. 4:**
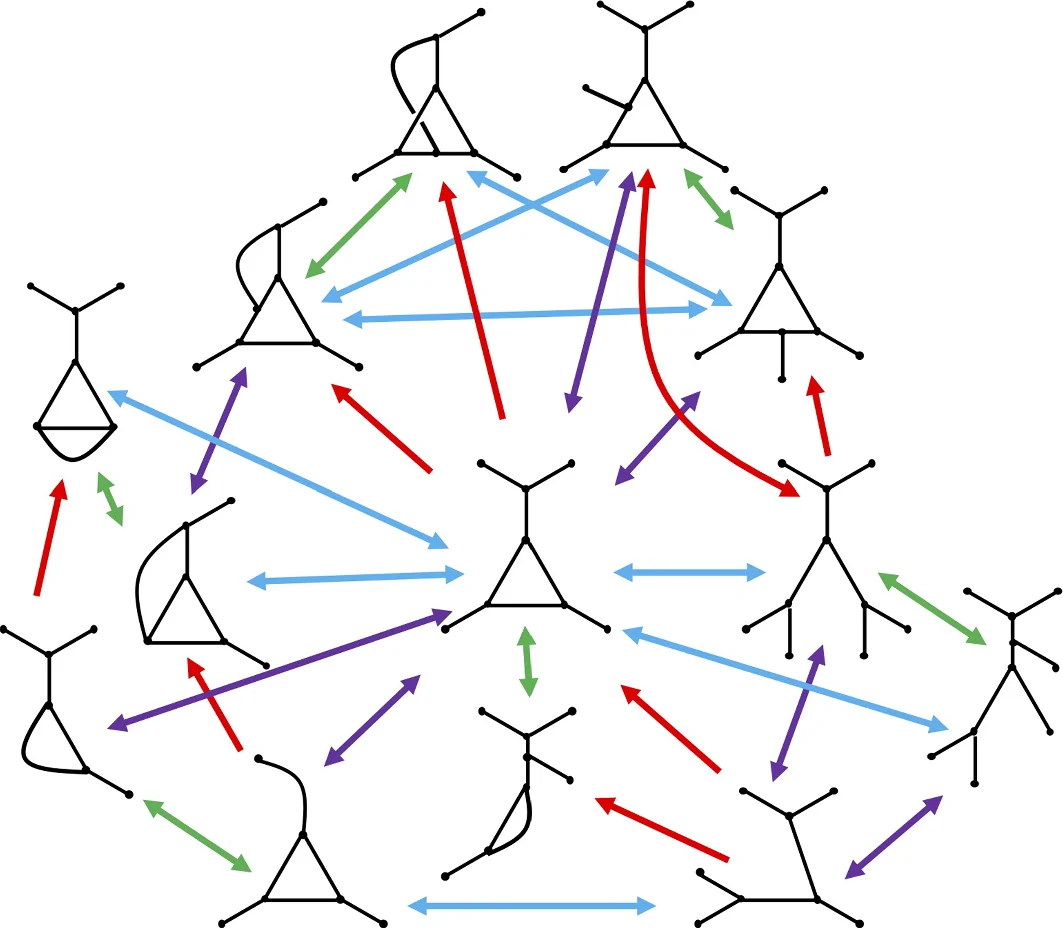
Example of the space of mitochondrial graphs showing a non-exhaustive set of graphs that can be reached from an initial graph shown in the center by application of individual morphological operations. Arrows indicate types of morphological operations: blue - fission and tip-tip fusion; red - tip-side fusion; green - vertex flip; purple - branch outgrowth/resorption. Arrows are also included to show transitions among the graphs that can be reached from the initial graph (Color figure online)

Second, in many cases a given morphological operation can be applied to a single graph in multiple ways. For example, fission could be applied at many different edges. Consider the example shown in Fig. 4, which shows a single mitochondrial graph together with some of the mitochondrial graphs that can be obtained from the first one by applying single morphological operations like fission, fusion. etc. This complicated figure is only showing a fraction of the graphs that can be obtained in this way from the first graph, and a similarly complex picture exists for every possible mitochondrial graph. Finally, there is an added complication due to the challenge of deciding when two graphs are isomorphic, which makes enumerating all the graphs very difficult computationally. The diagrams that result are not just complicated, they become impossibly messy when projected down into a two dimensional plot for humans to visualize, resulting in a "hairball" that provides no insight.

Because of the visualization challenges and also due to the size of the space MG compared to the size of experimentally realistic datasets, the main approach to characterizing the statistical distribution of mitochondrial graphs in the past has been to apply graph theoretic descriptors like diameter or cyclomatic number to summarize individual graphs, and then perform statistical analysis on those descriptors. Here we propose to apply a different approach using a framework from abstract algebra, to develop a simplified representation of the space of mitochondrial graphs based not on comparison of the graphs themselves, but based on the operations that convert one graph into another. Algebraic graph theory has seen only very limited application in biological problems, and has not to our knowledge been used to understand mitochondrial architecture. We believe that this work will furnish new mathematical problems while, at the same time, providing a new way to get at a fundamental biological question by making it possible to visualize and compare distributions of mitochondrial graphs between different cell types, mutants, growth conditions, and disease states.

## Results

### State Space of Mitochondrial Graphs is Irreducible

**Fig. 5:**
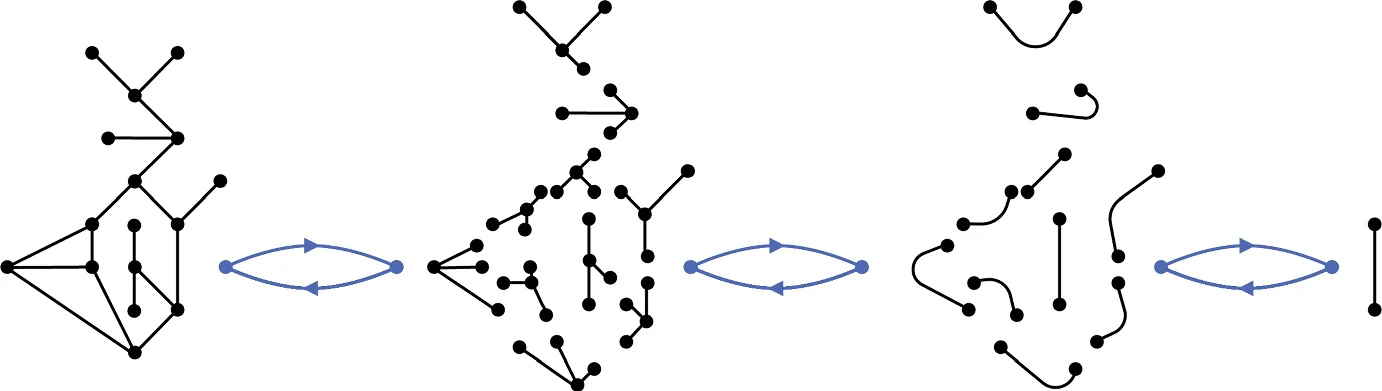
Visual explanation of how it is possible to convert from any mitochondria graph to a single mitochondrion and vice versa (Color figure online)

We propose to represent the space of mitochondrial graphs *MG*, not in terms of the vast space of possible graph structures, but in terms of the set of morphological operations that convert one graph to another. To do this, it is necessary that the space of possible graphs be irreducible with respect to this set of transformations. We want to show that it is possible to find a sequence of the morphological transitions listed in Fig. 2 that would allow us to interconvert between any two mitochondria graphs. As shown in Fig. 5, for any mitochondria graph, we can perform a series of fissions to transform it into a collection of Y-shaped or I-shaped tubules. Then, we can resorb one branch on each Y-tubule into an I-shaped graph, a mitochondria graph with only two degree-one vertices (i.e. $K_2$), and tip-to-tip fuse the remaining connected components into a single mitochondrion. Since all of these transitions have inverses, fission being the inverse of tip-to-tip fusion and outgrowth the inverse of resorption, we can reverse this process to reach any other possible mitochondria graph. We recognize, of course, that in actual cells this sequence of events is highly unlikely to occur, but it is not impossible, and therefore we are justified in invoking irreducibility in derivations when necessary.

This demonstration seems to involve a highly unlikely set of sequential fissions, but mitochondria have been observed to adjust their morphology similarly to the aforementioned thought experiment. The mitochondrial matrix has been observed to temporarily contract into nodules in a way that visually resembles a network fissioning into many smaller connected components – although the network remained connected by thin, almost invisible, tubules (Sturm et al. 2025). Surprisingly, Lee and Yoon (2014) found that fission deficiency greatly amplified this phenomenon. This compaction into a chain of nodules is at least visually reminiscent of the proposed reduction to I-tubules. Moreover, this interconvertibility is a property of groups and several other types of algebraic structures, suggesting that there perhaps are hidden symmetries in mitochondrial dynamics that could provide a new way to view relations between mitochondrial networks based not only on their traditional graph-theoretic properties but on relations between the transformations that produce them. The idea is to define an algebraic structure that describes how different morphological operations compose together, and then view the effect of these operations as actions on the set of mitochondrial graphs. In this viewpoint, the interconvertibility property would correspond to a transitive action.

Mitochondrial dynamics offer a promising avenue to apply abstract algebra to raise questions of interest for both mathematics and cell biology. The key properties that one uses to define an algebraic structure have biological interpretations that could lead to meaningful insights. For instance, the interconvertibility property of groups arises from the requirement that each element must have an inverse. Mitochondria are interconvertible, however, tip-to-side fusion does not have a direct inverse. Rather, as seen in Fig. 2, tip-to-side fusion has a functional inverse that (in most cases) is obtained by first performing resorption followed by fission. In algebraic terms, this functional inverse is a relation (Shahriari 2017). As implied in our interconvertibility result, it is possible to interconvert between any two mitochondria graphs without tip-to-side fusion – it can be replaced by composing tip-to-tip fusion and outgrowth.

But tip-side fusion is not a rare event. In their investigation of the mechanisms behind mitochondrial fusion, Gatti and colleagues (Gatti et al. 2023) observed that about 75% of fusion events were tip-to-side. Furthermore, they found evidence suggesting that tip-to-side and tip-to-tip fusion are mechanistically different: actin was present in about 88% of tip-to-side fusion events but only in 50% of tip-to-tip fusion events, arguing that a different molecular machine may have evolved for tip-side fusion. This example characterizing tip-to-side fusion demonstrates how the exercise of defining an algebraic structure on the mitochondrial dynamics state space encourages us to consider mitochondrial biology from new perspectives.

### An Equivalence Relation Based on Mitochondrial Dynamics

The big challenge with cell representation in general, is the large size of the possible space of cell structures. This is exemplified by the problem of trying to enumerate the space of possible mitochondrial graphs. As the graph becomes large, the number of possible graphs, as well as the number of distinct graphs that can be produced from a given graph by a set of transformations, grows combinatorially. For example, it is often possible to apply a particular operation such as fission to many possible edges in a graph to produce different graphs as an outcome. One way to simplify this problem is to find some criterion by which collections of mitochondrial graphs can be viewed as equivalent.

We will show that the vertex flip can uniquely be used to define an equivalence relation. We begin with the following definition.

#### Definition 2.1

We will say that mitochondria graphs *M* and $M^{\prime }$ are vertex flip interconvertible if there exists a sequence of vertex flips taking *M* to $M^{\prime }$ and vice versa.

In contrast to the other operations, vertex flip does not alter the number of degree 1 or 3 vertices, hence for two graphs to be vertex flip interconvertible, the number of degree 1 and 3 vertices must be the same for both graphs. We can keep track of the number of these quantities with the vector M=[p,n], in which *p* and *n* are the number of degree 1 and degree 3 vertices respectively. Before exploring how morphological operations work in this representation, we note that there are constraints on this representation such that only certain combinations of *p* and *n* are possible. First, the number of vertices *p* or *n* cannot be less than zero. Second, given the value of n, the value of p must obey defined bounds:

#### Proposition 2.1

Let [*p*, *n*] be the vector representing a single connected component of a mitochondria graph, then the maximum value of *p* is n+2 and the minimum is 0 if *n* is even and 1 if *n* is odd.

#### Proof

1. (Upper Bound) Consider the induced subgraph Y⊆M, where the set of vertices $N_Y$ of *Y* are all the degree-three vertices in *M*, and $n = |N_Y|$. Note that the degree of a vertex in *Y* is not always three, but can be one, two, or three depending on the other vertices in *Y* it’s connected to. Since there are *p* degree-one vertices in *M* which collectively are adjacent to the set of *n* vertices in *Y*, it follows by definition of *M* and *Y* that $3n = p+\sum _n\deg (y_n)$. By the Handshaking Lemma, we have $p = 3n - 2|E_Y|$. Hence to maximize *p*, we must minimize the number of edges in *Y*. From Proposition 1.3.13 in West (2001), the minimum is n-1. Thus, the maximum of *p* is p=n+2.
2. (Lower Bound) Consider the cases in which n=1 and n=2. For the n=1 case, it follows from Proposition 1.3.5 in West (2001), that we must have an even number of odd-degree vertices, and hence we must have another odd-degree vertex that is not a degree-three vertex. Hence the minimum number of degree-one vertices for the n=1 case is p=1 (this graph would have a single self-loop and be shaped like the letter “P"). For the second base case, n=2, we can reach it by performing outgrowth on the graph given by n=1, and as a consequence, we must produce a new degree-one vertex. We can then fuse the two degree-one vertices together into a single edge resulting in a graph with p=0 degree-one vertices for the n+1 case. We can inductively continue this process of performing outgrowth, and then performing tip-to-tip fusion as soon as two degree-one vertices are available..

□

Second, we note:

#### Proposition 2.2

For any connected component of a mitochondria graph [*p*, *n*], *p* can be any element of the set $p\in \{0,2,4,\ldots ,n, n+2\}$ if *n* is even and $p\in \{1,3,5, \ldots ,n, n+2\}$ if *n* is odd.

#### Proof

Consider the base case with p=0 if *n* is even and p=1 if *n* is odd. We can perform fission on an edge while maintaining a single connected component to get [p+2,n]. We can repeat this process until we get [n+2,n], at which point we can’t maintain a single connected component. □

Now we can return to the concept of vertex-flip interconvertibility. The following proposition shows that all single-component graphs with the same number of degree-three and degree-one vertices are vertex-flip interconvertible.

#### Proposition 2.3

Let *M* and $M^{\prime }$ both be single-component mitochondria graphs with the vector representation [*p*, *n*], then *M* and $M^{\prime }$ are vertex flip interconvertible.

#### Proof

We would first like to note that it is cumbersome to define a vertex flip without using a picture. To make this proof easier to understand and follow, we will rely heavily on pictures without using any other definition of a vertex flip. Our proof relies on induction, and we have two mitochondria graphs which serve as base cases:

- The vector [0, 0], is the null mitochondria graph which corresponds to a toroidal shaped mitochondrion. It is vacuously vertex flip interconvertible.
- The vector [2, 0] represents only one possible mitochondria graph, an I-tubule. It is vacuously vertex flip interconvertible.

**Fig. 6:**
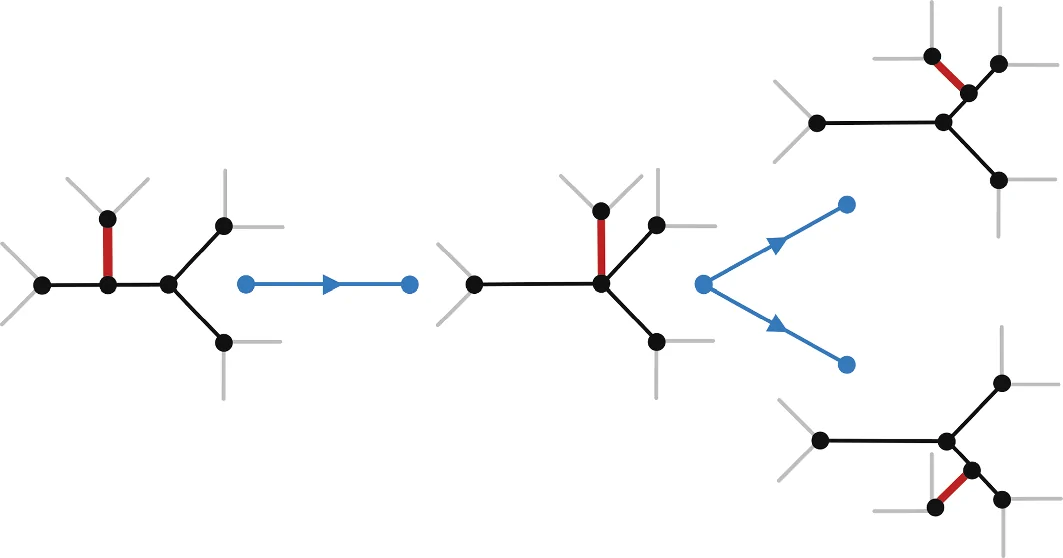
Image showing how it is possible to move the red colored edge next to any other edge using only vertex flips by “sliding" it along its “neighboring" edge (Color figure online)

Assume inductively that all graphs with a single connected component described by [*p*, *n*] are vertex flip interconvertible. Notice in Fig. 6 that we can move one pendant edge (an edge that connects a degree 3 vertex to a degree 1 vertex) next to any other edge with just vertex flips by “sliding" the pendant edge along its “neighboring edge".

Since we get [p+1,n+1] by outgrowing a pendant edge from [*p*, *n*], and can get back to a [*p*, *n*] graph by resorbing a pendant edge, then all graphs [p+1,n+1] are vertex flip interconvertible. Moreover, we can obtain [p,n+2] from [*p*, *n*] by inserting a new edge such that it connects two edges (by performing two outgrowth steps followed by a tip-to-tip fusion step).

**Fig. 7:**
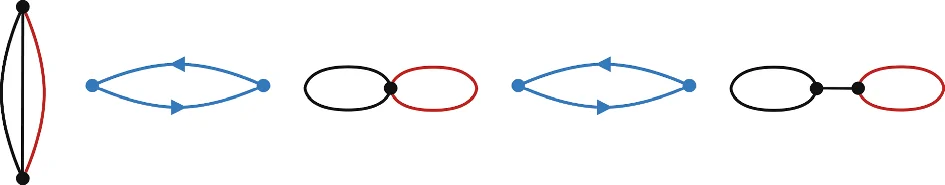
Image showing how both the mitochondria graphs represented by [0, 2] are vertex flip interconvertible (Color figure online)

As shown in Figs. 6 and 7, we can move this edge next to any other edge using only vertex flips by “sliding" the edge along its neighboring edges. Hence all graphs [p,n+2] are vertex flip equivalent. It follows from induction and proposition 2.2 that the set of graphs corresponding to any [*p*, *n*] are vertex flip interconvertible. □

Finally, we note several properties of vertex flip interconvertibility.

1. First, it is straightforward to show that any graph is vertex flip interconvertible with itself.
2. Second, if graph $M^{\prime }$ can be reached from graph *M* by a sequence of vertex flips, then the converse is also true.
3. Third, if graphs *A* and *B* are vertex flip interconvertible, and graphs *B* and *C* are vertex flip interconvertible, then so are graphs *A* and *C*.

Because the relation of vertex flip interconvertibility is reflexive, symmetric, and transitive, it is an equivalence relation, hence the set of all graphs that are vertex-flip interconvertible to any given graph constitute an equivalence class. Each distinct vector [*p*, *n*] defines one such equivalence class, which taken together partition the space of possible single-component mitochondrial graphs. We note that none of the other operations of mitochondrial dynamics (fission, fusion etc) satisfy the symmetry property needed to define an equivalence relation.

### Representing Mitochondrial Dynamics of a Single Component

We now consider how the vertex flip equivalence relation allows us to simplify the space of mitochondrial networks and their dynamics, by focusing on the special case in which the mitochondrial network is a single connected component. This is not just an arbitrary abstract notion - in many cell types, the mitochondrial network is in fact dominated by a single component (Mostov et al. 2026). If we consider only mitochondrial graphs with a single component, and only allow those morphological operations that retain a single connected component, we can define a category in which the objects are the vertex flip equivalence classes and the morphisms correspond to the morphological operations that convert one mitograph to another.

#### Definition 2.2

The Category *mitograph*, which we denote as *mG*, has as objects $\text {Ob}(mG)$ the sets of vertex flip equivalent graphs, and the morphisms of *mG* are the morphological transitions of mitochondrial dynamics that convert a graph in one vertex flip equivalence class to a graph in another.

First we need a way to name the individual equivalence classes. As will be discussed below, there is more than one way to do that, but here we continue to use our vector notation [*p*, *n*]. In this way we can label all the members of *Ob*(*mG*).

**Fig. 8:**
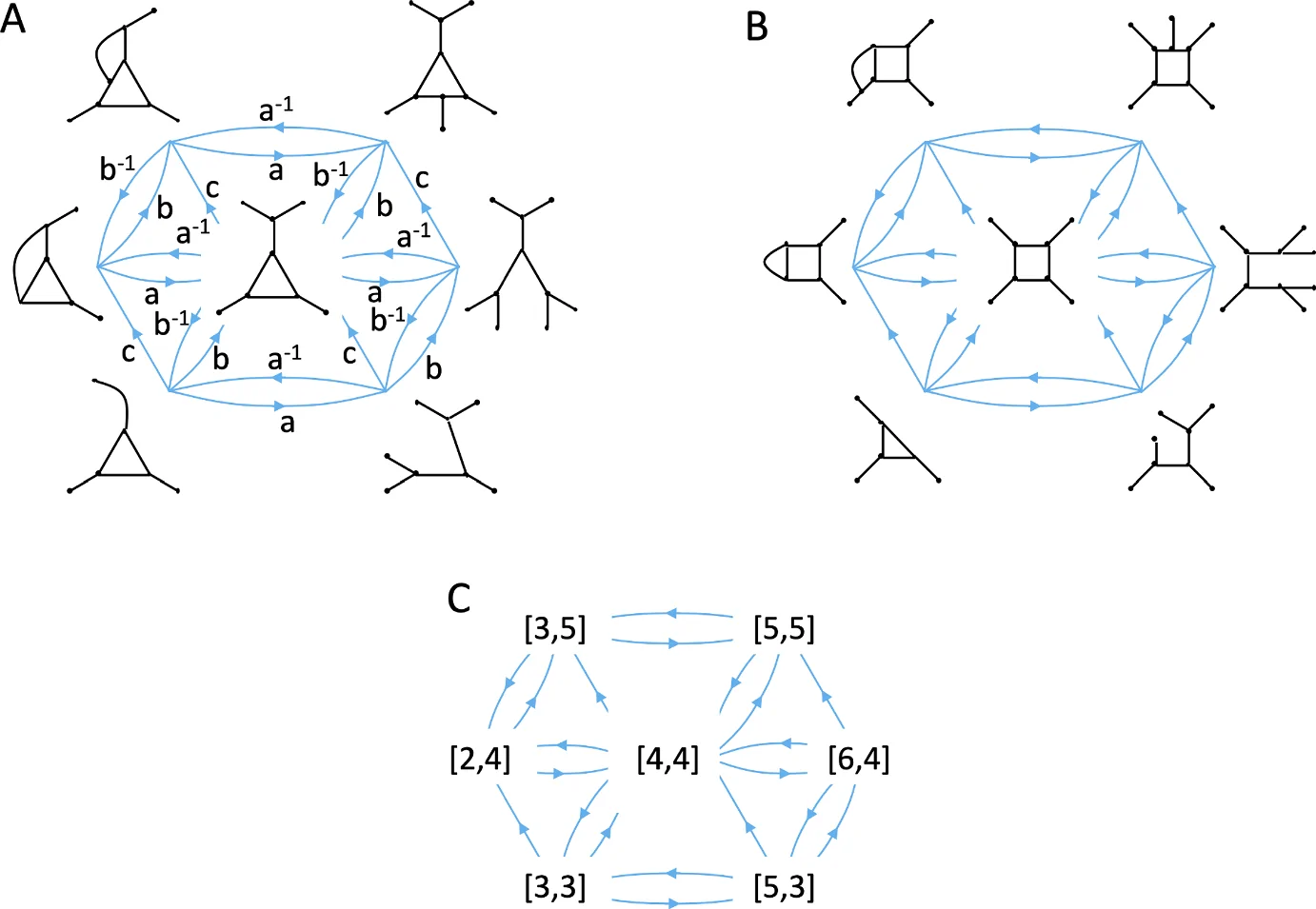
Any morphological operation applied to a pair of vertex flip-equivalent graphs yields a pair of graphs that are vertex-flip equivalent to each other (Color figure online)

To define the morphisms, we want to represent the actions of the morphological operations defined above. When applied to the set of mitochondrial graphs, it is not sufficient to specify one of the operations $$\{a,a^{-1}, b, b^{-1}, c, d, e\}$$, but also to define how it is applied to any given graph. For instance, we can have an element $a:M\to M^{\prime }$ that fissions a specific edge in *M* to create $M^{\prime }$, but we can also have another element $a:M\to M^{\prime \prime }$ that creates a different graph by performing fission on a different edge. The situation becomes greatly simplified if we consider how these operations act on vertex flip equivalence classes. In this case, we note that the action of any operation on vertex-flip equivalent graphs produces vertex flip equivalent outputs, as illustrated in Fig. 8.

Using our vector notation we can then define each morphism corresponding to the morphological transformation simply as the addition of constant vectors:

$$\begin{aligned} a = \begin{bmatrix} 2\\ 0 \end{bmatrix},\quad a^{-1} = -a, \quad b = \begin{bmatrix} 1\\ 1 \end{bmatrix},\quad b^{-1} = -b,\quad&c = \begin{bmatrix} -1\\ 1 \end{bmatrix} \end{aligned}$$

Composition of morphisms is thus achieved by vector addition. With this representation we can represent sequential changes in the graph structure of real mitochondria observed in live-cell images in terms of sequential vector addition operations. Using these operations it is possible to describe a mitochondria graph as the sum of transitions through which it can be reached from some reference point, say the graph represented by [2, 0] (which we will denote as *I*). For instance, we can represent a mitochondria graph as the vector $$\begin{bmatrix} 2, 4 \end{bmatrix}$$ or as I+2b+2c.

To construct a category we take these operations as the generating morphisms, with the null operation e serving as the identity morphism. We take all morphisms to exist between any source and target objects that are permitted for mitographs. Because mitographs cannot have a negative value for p or n, morphisms cannot have a target object for which p or n is negative. Due to the constraint from Proposition 2.1 that p≤n+2, no morphism can have a source or target that violates this constraint. Otherwise, it is always possible to apply any of the morphological operations to one or more graphs contained in any given equivalence class, provided the constraints on mitograph structure are respected.

To confirm that *mG* defined in this way really is a category, we only need to show that every object has an identity morphism and that the morphisms are composable. The identity morphism is just null operation which we have named e, which takes each graph to itself. Composability is guaranteed because we have already restricted morphisms to only include permissible transitions.

**Fig. 9:**
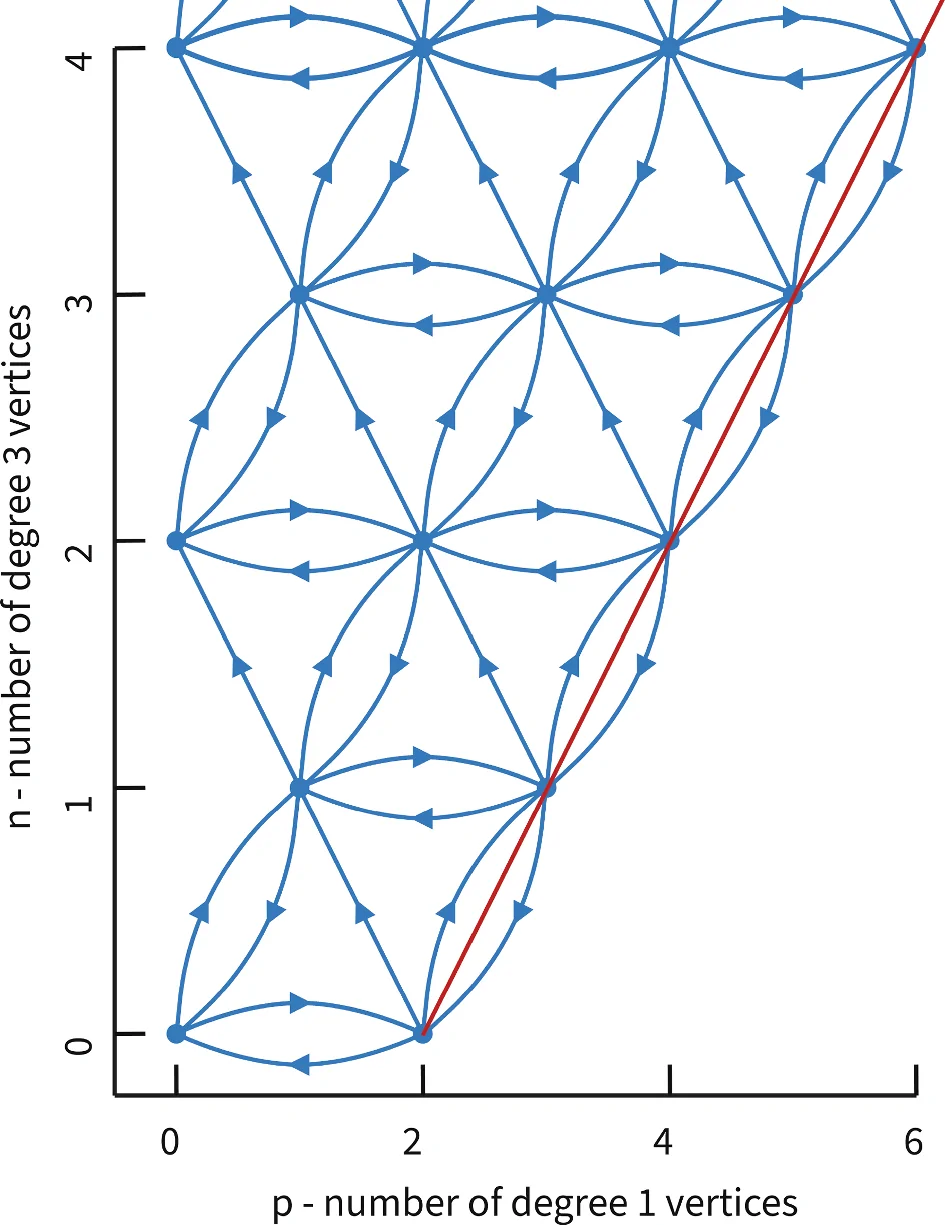
Structure of *mG*. Dots represent vertex-flip equivalence classes of single-component mitochondrial graphs, linked by transitions representing the morphological operations that generate *mG*. Red line signifies the upper bound on p given n, corresponds to mitochondrial graphs that are trees. The left boundary at p=0 corresponds to the cubic graphs, with the exception of $\left[ 0,0\right]$ being the null graph that represents a toroidal mitochondrion (Color figure online)

The quiver diagram (which shows the objects as dots and the generating morphisms as arrows ) of *mG* is described in Fig. 9. The visually simple structure of *mG* arises from the fact that each of the morphisms applied to an object labeled with a vector [*p*, *n*] as the source, has as its target an adjacent object when the objects are arranged according to their vector representation. The boundary on the right is given by the constraint p≤n+2. In this representation, we gain the ability to easily map observed graphs onto objects of *mG* just by counting vertices of degree 1 and 3.

By using *mG* to represent the structure of mitochondrial dynamics, we gain mathematical tractability as well as a straightforward way to map observed data onto the representation, but we do so at the cost of making an apparently extreme assumption that the mitochondrial network consists of a single connected component. This may at first seem to be a drastic assumption, but a combinatorics argument shows that the features of mitochondrial graphs make the presence of a single giant component likely (Mostov et al. 2026). And in actual fact, budding yeast mitochondria often do take the form of a single large connected component plus some number of I tubules (Viana et al. 2020). A similar situation has also been seen in other cell types (Zamponi et al. 2018). These are all still multi-component graphs. However, in such cases, fusion with an I tubule would either be by tip-to-tip fusion, in which case it would simply add onto the end of a pendant edge and thus have no effect on the graph structure of the giant component, or else it would fuse with the giant component by tip-side fusion in which case it would mimic branch outgrowth which is already one of the elements of *mG*. A fission operation that produces a new I tubule would have to have involved a pendant edge, and leaves behind a pendant edge in the same position, so again it would not affect the graph structure of the giant component. So, the presence of a collection of I tubules along with a single giant component has no effect on the algebraic structure of the transformations operating on the single giant component. Thus, depending on cell type and growth condition, the single-component mitochondrial graph category *mG* may in fact be a biologically reasonable approximation of real mitochondrial dynamics.

### Alternative Labelings for *Ob*(*mG*)

In our development of the representation above, we have labeled the vertex flip equivalence classes using a vector [*p*, *n*] based on the degree sequence (i.e. counting the number of vertices of degree 1 and 3, with all other degrees being disallowed. Our choice of the degree sequence as a basis for labeling the vertex flip equivalence classes was somewhat arbitrary, based only on the use of the degree sequence for certain types of extremal graph theory questions. There are, however, other ways that these same equivalence classes could be labeled. The cyclomatic number r, while defined in terms of cycle bases, can be expressed in the formula r=E - V + C where E is the number of edges of a graph, V the number of vertices, and C the number of components. Because vertex flip does not change the number of edges or vertices, and only acts on a single component, it does not change r. We therefore consider a representation in terms of r and E, which we denote (*r*, *E*). The reason for choosing these two values, in addition to the fact that they are unchanged by vertex flip, is that they characterize biologically relevant properties of the mitochondrion, with *r* capturing the interconnectedness in terms of loops, and E capturing a measure of network size. We prefer E over the number of vertices as a network size measure, because the biomass of the mitochondrion is contained in the edges.

**Fig. 10:**
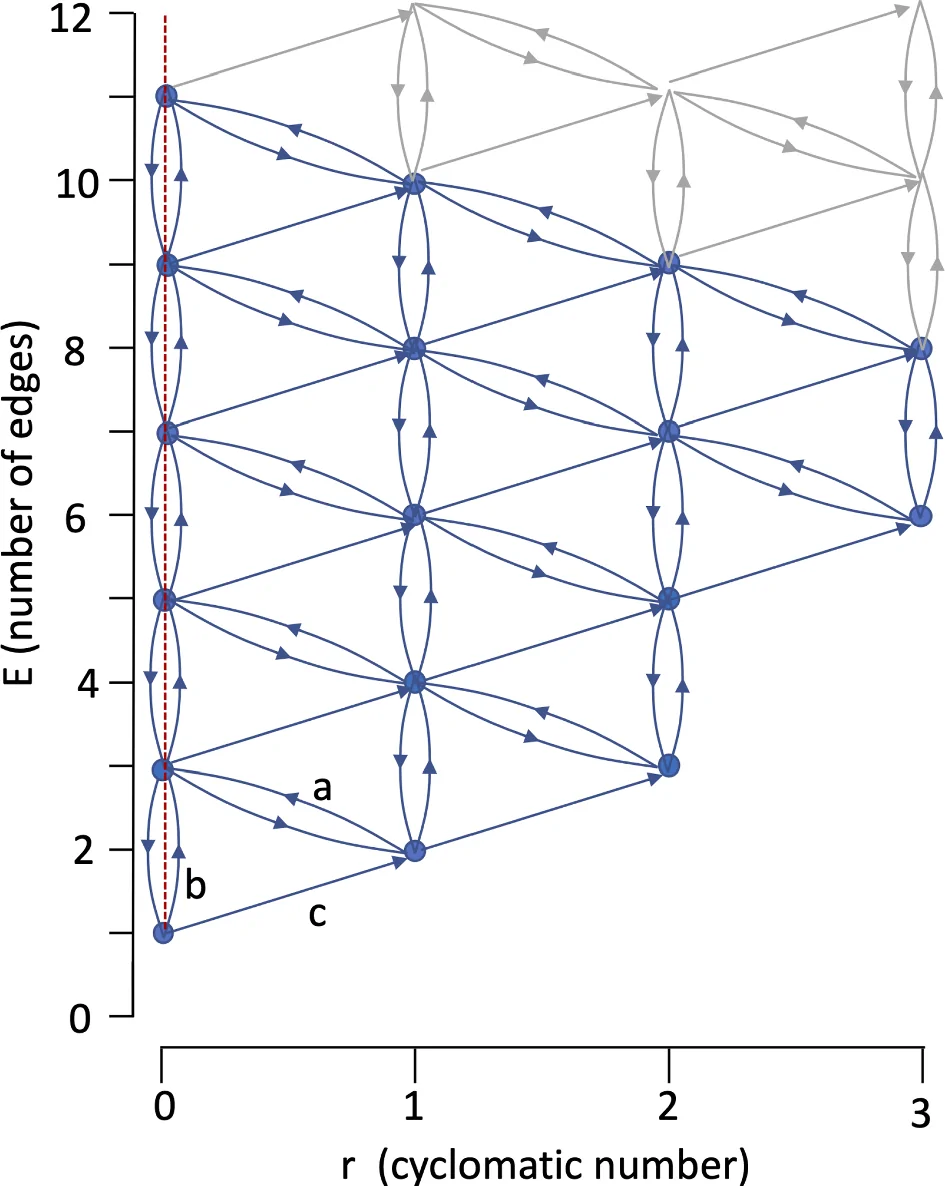
Alternate labeling of vertex flip equivalence classes using cyclomatic number r and total number of edges E. Red line indicates the corresponding classes marked by the red line in Fig. 9 (Color figure online)

We can also define the morphological operations in terms of how they change r and E, which gives us the diagram shown in Fig. 10. In this diagram, branch outgrowth and resorption leave the cyclomatic number unchanged and just increase or decrease E. The left edge of the diagram contains the trees. The lower boundary of the diagram is determined by the minimum number of edges required to have a graph with a particular cyclomatic number. As shown in Methods, this is given by the (*r*, *E*) values (0, 1), (1, 2), (3, 3),..., (3r-3).

This representation is really just a re-labeling of the vertex flip equivalence classes previously labeled as [*p*, *n*]. To see this, we can express r and E in terms of p and n as derived in Methods:

$$\begin{aligned} r = \frac{n-p}{2} + 1 \end{aligned}$$

$$\begin{aligned} E = \frac{3n+p}{2} \end{aligned}$$

We can also find the inverse relations:

$$\begin{aligned} p = \frac{E-3r+3}{2} \end{aligned}$$

$$\begin{aligned} n = \frac{r+E-1}{2} \end{aligned}$$

These two sets of equation show that the representation in terms of [*p*, *n*] is isomorphic to the representation in terms of (*r*, *E*).

**Fig. 11:**
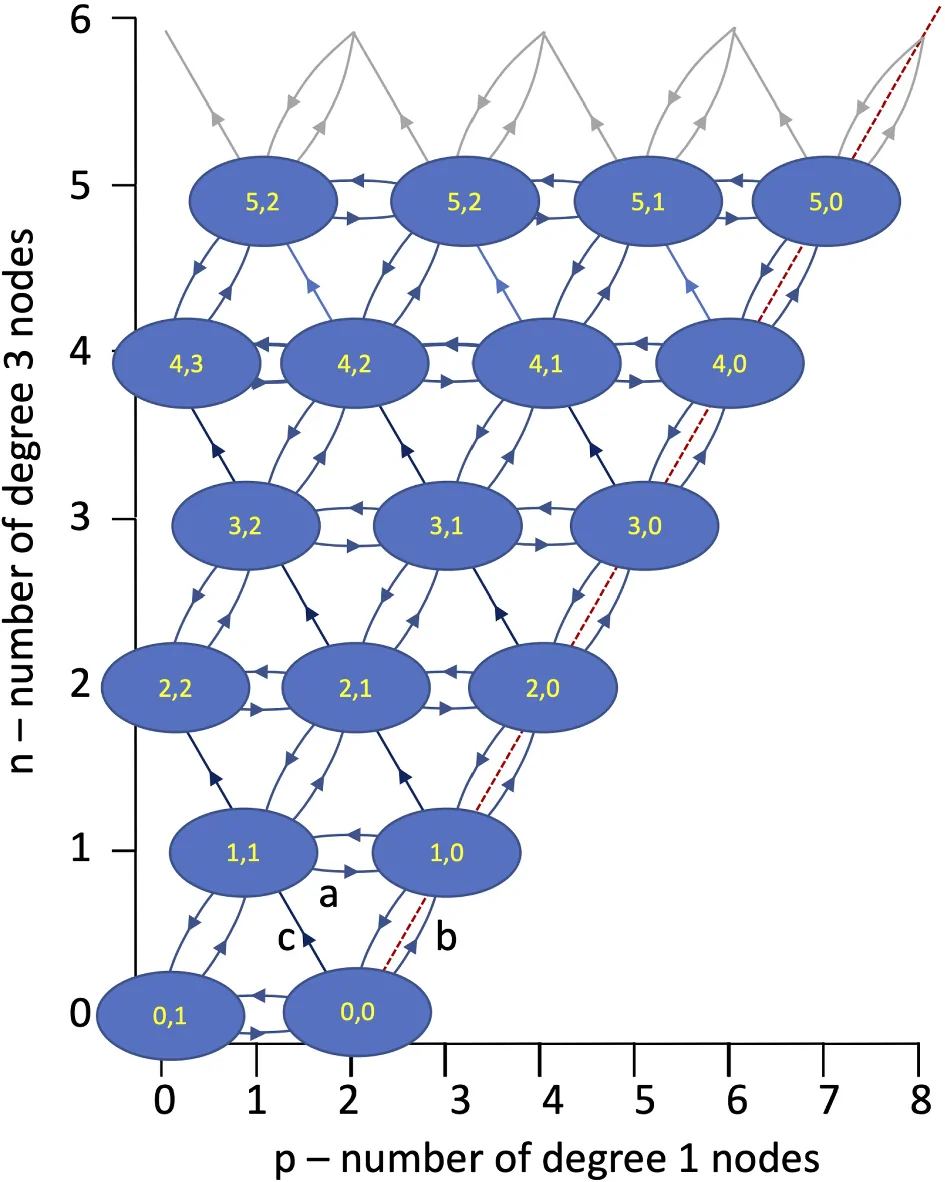
Labeling equivalence classes based on the minimal number of branch outgrowths and tip-tip fusions (Color figure online)

One way of labeling that is particularly convenient for computational applications is to assign the label (0, 0) to the I-tubule ($K_2$, defined in [*p*, *n*] labeling as [2, 0] or in (*r*, *E*) labeling as (0, 1)) and then label all equivalence classes based on the minimum number $n_b$ of branch outgrowth operations and $n_a$ of tip-tip fusion operations required to reach a given equivalence class starting from the I-tubule, to produce a label $(n_b,n_a)$. This labeling scheme is illustrated in Fig. 11. The advantage of this scheme is that the numbers used for labels differ by one between neighbors, rather than by intervals of 2 in the other schemes. This means that if this representation is used to record counts of real data, the data will occupy a regular grid (albeit not a rectangular one) greatly facilitating computation of neighbor differences, spatial correlation functions, etc. All of this would of course be possible with the other representations, but most likely would entail converting them into something like the $(n_b,n_a)$ notation in Fig. 11 as an intermediate step.

Because these three ways of labeling produce isomorphic structures, it does not matter which one we use, and it may simply depend on which labeling strategy seems more intuitive in a given situation. For example, labeling based on degree sequence, i.e. [*p*, *n*], is a purely local feature of the individual vertices and is easy to determine from a graph just by visually counting vertices, whereas cyclomatic number tells us more about connectivity of the graph and can simplify some proofs but takes more work to calculate from the image of a graph. Moreover, these three ways of labeling the vertex flip equivalence classes are by no means the only ones possible. One could for example use the total number of vertices and total number of edges.

In fact, we don’t even need two numbers to label the objects of *mG*, because they are countably infinite. Starting from the lower left of Fig. 9, we can traverse the lattice row by row and column by column, assigning increasing integers to each object in turn. As derived in Methods, the label for class [*p*, *n*] can be given in closed form as:

$$\begin{aligned} label = 2n + \lfloor \frac{n-1}{2} \rfloor \lfloor \frac{n}{2} \rfloor + \lfloor \frac{p}{2}\rfloor \end{aligned}$$

By replacing a pair of labels like p and n with a single integer label, this representation allows the distribution of graphs across the [*p*, *n*] classes to be compactly stored as a one-dimensional vector, which may have advantages for analyzing large numbers of different cell types or mutants using tools like PCA. But in general, such a representation is much harder to visualize compared to either [*p*, *n*] or (*r*, *s*), for two reasons. First, because the neighborhood relation present in the lattice diagrams would be broken up in the linear representation, and second, because the morphological operations would represent jumps back and forth along the line of integers.

Although we can come up with many other examples of labelings that all give an isomorphic structure, this is not going to be the case for any arbitrary graph descriptors. For example, vertex flip equivalent graphs can have different graph diameters, so if diameter is part of a labeling scheme, it will not give an isomorphic structure to the space.

### *mG* Contains the Automorphism Group of Each Mitochondrial Graph

The mitochondrial structure category *MG* described here is evidently a complex structure. One approach to understanding such a system is to look for structures that contained within the larger structure. A standard approach with complicated algebraic structures is to focus on transformations that take an object back to itself. In some cases, these sets of transformation may constitute a group, in which case approaches from group theory can be used to classify the structure of the group, thereby gaining a foothold on the more complex structure of the whole system.

Consider all the transformations (viewed as sequencers or strings of morphological operations) that start with some mitochondrial graph *G* in *MG* and after some number of transformations to other graphs, ends up producing the original graph G. For any such transformation, its inverse is also part of the set. When only transformations from G to G are considered, the product becomes a binary function, unlike the general case of transformations between graphs. All other properties (associativity, inverse, identity) are unchanged, and hence the set of such transformations constitutes a group. One way to characterize the structure of *MG* will, thus, be to characterize these groups of return transformations for each mitochondria graph *G* of *MG*. Since different groups correspond to different types of symmetries, identifying the automorphism group for a graph is a standard way of learning about symmetries that may be present within the graph (Godsil and Royle 2001 ). From a biological viewpoint, symmetry within a graph would indicate that the same set of connections would be observed as one moves out from two or more different vertices, and could potentially reflect symmetry in transport or other processes within the network.

We therefore consider the set $Q_G$ of strings of transformations (i.e. morphisms of *MG* generated by the morphological operators) *q* that start and end at a particular mitochondria graph *G*. If we label each vertex in *G*, then some choices of *q* could permute the vertex labels. To properly define a group on $Q_G$, we would have to determine how each morphological transformation affects the vertex labels of a mitochondria graph. Some transformations, like fission and outgrowth, add new vertices, while others, like tip-to-tip fusion and resorption, remove them. The following proposition shows a procedure for adding and removing vertex labels in such a way that any choice of *q* would not add or remove vertex labels from *G*, but rather just permute the labels.

#### Proposition 2.4

Let *G* be a a mitochondria graph with *N* vertices, and consider the following labeling scheme:

- Label the vertices with natural numbers 1 through *N* by arbitrarily assigning the labels.
- Whenever we add a vertex to *G*, we label the new vertex N+1.
- Whenever we remove a vertex numbered *k* from *G*, if k<N, then we relabel vertex *N* to *k*.

Furthermore, let $Q_G$ be the set of strings of transformations that start and end at *G*. Then $Q_G$ is the permutation group on *N* elements, and, contains the automorphism group of *G*, defined as the set of all permutations of vertices that preserve the edge structure of *G*.

#### Proof

Let *G* be a mitochondria graph with *N* vertices. Taking note of our explanation of how mitochondrial dynamics are fully interconvertible back in section 2.1, we can first convert *G* into a graph $G^{\prime }$ that consists of *N* I-tubules such that each I-tubule contains a vertex $$k\in \{1,\ldots , N\}$$ connected to a vertex k+N.

Next, consider the vertices labeled *i* and *j* in $G^{\prime }$ where i,j≤N. If we want *i* to be a pendant vertex and a neighbor of *j* in the final graph, then we tip-to-tip fusion vertices i+N and j+N together. Else, if we want *i* to be a degree-three vertex and a neighbor of *j*, then we can tip-to-side fusion *i* to the edge connected to *j*. Iterating this process across all *N* of the I-tubules in such a way that we end back up at the graph *G*, we see it is possible to reach any relabeling of *G*. Thus, there are no “illegal configurations" in $$Q_G$$, and $$Q_G$$ is the permutation group on *N* elements, which must contain the automorphism group of *G* as a subgroup (Godsil and Royle 2001). □

Now, if instead of individual graphs, we consider *mG* and examine the set of morphisms generated by the base morphisms corresponding to the morphological operations, that return a class [*p*, *n*] back to itself. These again constitute a group. These groups will be different for different equivalence classes because the constraints on valid classes (see for example Fig. 9 ) mean that some strings of transformations that can be applied to one class M cannot be applied to another without producing an invalid [*p*, *n*]. But for all the graphs contained within a given object of *mG*, the argument above shows that the set of morphisms from the object back to the same object, when acting on any specific graph within that object, contain the automorphism group of that graph.

**Fig. 12:**
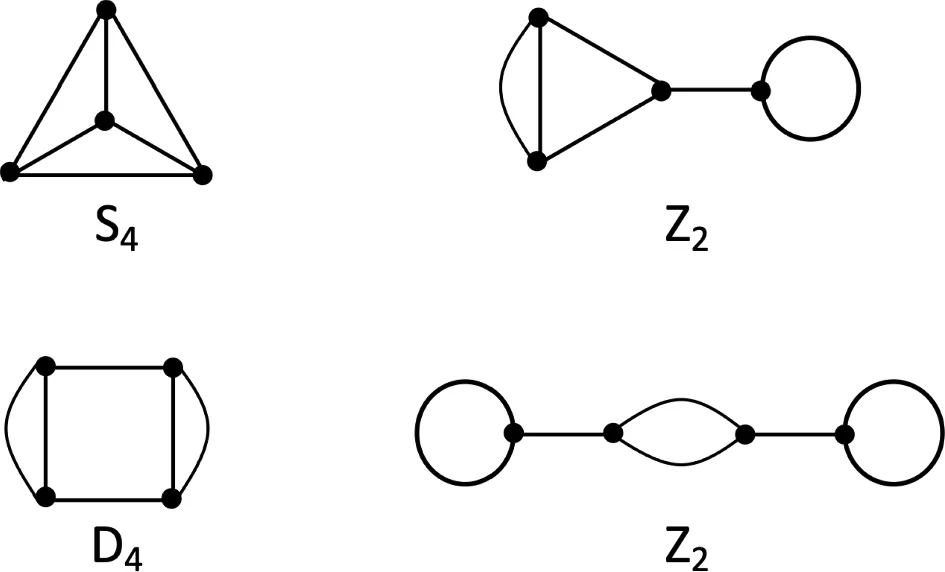
The four graphs represented by vector [4,0] with their corresponding automorphism groups, showing that they are not all the same

The most basic question is whether the automorphism groups of the graphs corresponding to a particular class [*p*, *n*] can tell us anything about the structure of the group of return transitions for the class. This would be the case if, for example, all graphs in a given vertex flip equivalence class had the same automorphism group. But it is easy to show by examples that this is not the case. A simple example is provided by the vector $\left[ 4,0\right]$ which includes the complete graph $K_4$, for which the automorphism group is the symmetric group on four elements $S_4$, the square graph with two multiedges, for which the automorphism group is the dihedral group on a square $D_4$, as well as two other graphs, both containing self-loops, for which the automorphism group is the integers modulo two, $\mathbb {Z}_2$ (see Fig. 12). The difference among these automorphism groups reflects differences in the degree and type of symmetry within the graphs (Godsil and Royle 2001). For example, in $K_4$ all the vertices have the same relation to the rest of the graph so that any one can be interchanged for any other without changing the graph, which is why its automorphism group is $S_4$, the group of all possible permutations of the vertices.

### Extension to the Case of Multiple Components

We can take this representation further. If a mitochondria graph has multiple connected components, then we can represent each connected component with a vector, and the resulting multiset of vectors will be it’s own equivalence class.

#### Definition 2.3

A *multiset vector representation* of a general mitochondria graph is a multiset of vectors in which each vector counts the number of degree-one and degree-three vertices in a connected component. For instance,

$$\left\{ \begin{bmatrix}p_1\\ n_1 \end{bmatrix}, \begin{bmatrix}p_2\\ n_2 \end{bmatrix},\cdots ,\begin{bmatrix}p_j\\ n_j \end{bmatrix}\right\} $$

represents a mitochondria graph with *j* connected components.

For the multiset vector representation, we must define the transformations that change the number of connected components differently from those that don’t. For instance, if we have the graph representation $$\begin{bmatrix} 3, 3 \end{bmatrix}$$ and we want to perform fission on it, there would be two ways of doing so. We could fission a non-pendant edge to get the graph represented by $$\begin{bmatrix} 5,3 \end{bmatrix}$$, or we can fission a pendant edge to get $$\{\begin{bmatrix} 3,3 \end{bmatrix}, \begin{bmatrix} 2,0 \end{bmatrix}\}$$.

**Fig. 13:**
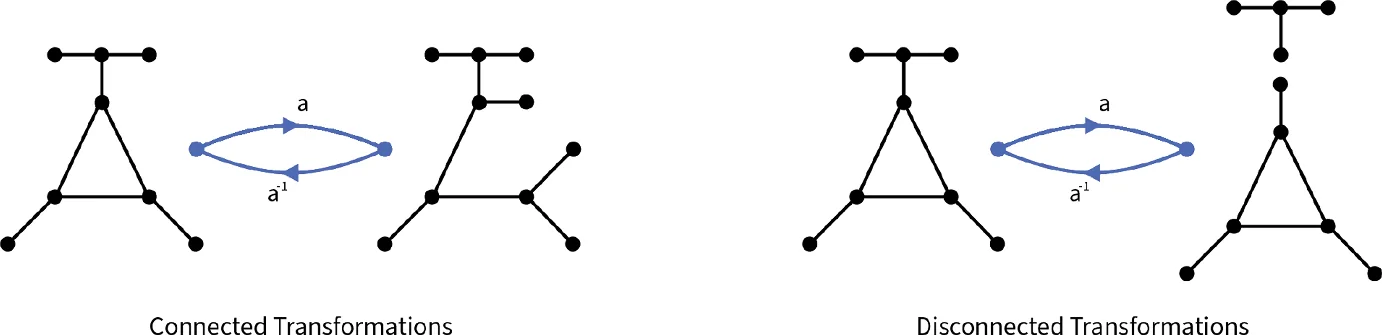
Comparison of connected fission/tip-to-tip fusion with disconnected fission/tip-to-tip fusion (Color figure online)

More generally, fission on any edge that is a cut-edge (an edge whose removal increases the number of connected components) would result in addition of a new vector to the multiset. We must therefore define a ‘connected fission’ $a_1$ which maintains the number of connected components, and a ‘disconnected fission’ $a_2$ which increases the number of connected components. Similarly, we must define connected and disconnected fusions (see Fig. 13).

The set of morphological operations defined on the space of vector multisets can then be represented as follows:

- *Connected Fission*: $$a_1(\begin{bmatrix} p,n \end{bmatrix}) = \begin{bmatrix} p+2,n \end{bmatrix}$$, where p<n+2.
- *Connected Tip-to-Tip Fusion*: $$a_1^{-1}(\begin{bmatrix} p,n \end{bmatrix}) = \begin{bmatrix} p-2,n \end{bmatrix}$$, where p≥2.
- *Disconnected Fission*: $$a_2(\begin{bmatrix} p,n \end{bmatrix}) = \{ \begin{bmatrix} p_1,n_1 \end{bmatrix}, \begin{bmatrix} p_2,n_2 \end{bmatrix}\}$$, where $n_1+n_2 = n$, $p_1+p_2 = p+2$, and $p_1,p_2\ge 1$. Clearly there are actually many different *a*2 based on how the n and p vertices are partitioned during the fission.
- *Disconnected Tip-to-Tip Fusion*: $$a_2^{-1}(\{ \begin{bmatrix} p_1,n_1 \end{bmatrix}, \begin{bmatrix} p_2,n_2 \end{bmatrix}\}) $$$$= \begin{bmatrix} p_1+p_2-2,n_1+n_2 \end{bmatrix}$$, where $$p_1,p_2\ge 1$$.
- *Outgrowth*: $$b(\begin{bmatrix} p,n \end{bmatrix})= \begin{bmatrix} p+1,n+1 \end{bmatrix}$$
- *Resorption*: $$b^{-1}(\begin{bmatrix} p,n \end{bmatrix})=\begin{bmatrix} p-1,n-1 \end{bmatrix}$$, where n,p≥1.
- *Connected Tip-to-Side Fusion*: $$c_1(\begin{bmatrix} p,n \end{bmatrix}) = \begin{bmatrix} p-1,n+1 \end{bmatrix}$$, where p≥1.
- *Disconnected Tip-to-Side Fusion*: $$c_2(\{ \begin{bmatrix} p_1,n_1 \end{bmatrix}, \begin{bmatrix} p_2,n_2 \end{bmatrix}\}) $$$$= \begin{bmatrix} p_1+p_2-1,n_1+n_2+1 \end{bmatrix}$$, where $$p_1\ge 1$$ and/or $$p_2\ge 1$$.
- *Mitophagy*: $$d(\begin{bmatrix} 2,0 \end{bmatrix}) = \emptyset $$, only possible on $$\begin{bmatrix} 2,0 \end{bmatrix}$$.

These operations are defined on individual vectors or pairs of vectors in a multiset, which are then replaced with the specified new vectors while all other vectors in the multiset are kept unchanged. No known mitochondrial morphological operations act on more than two components.

The restrictions on these transformations give us:

#### Definition 2.4

The category of *multiset mitochondrial graphs* *mmG* is the category in which $\text {Ob}(mmG)$ is the set of multiset vector representations of mitochondria graphs as defined above, and the morphisms of *mmG* are the transitions between $\text {Ob}(mmG)$. Furthermore, there are separate versions of $a_2$ morphisms defined for each possible way there is to split up a single connected component into two connected components.

Clearly the structure of *mmG* is much more complex than that of *mG* and cannot readily be visualized in a simple diagram. Because, as discussed above, a single giant component is the expected case for many cell types, we will not consider *mmG* further, and will focus only on *mG*.

### A Biologically-Inspired Distance Metric for Mitochondria Graphs

For many applications it is useful to be able to specify a measure of similarity between mitochondrial graphs. For example, such a similarity measure could be used to cluster data or to ask about how a population of observed graphs are distributed in the graph space. Statistical analysis of graph distributions requires methods to correct for correlations between highly similar objects. Many methods have been developed to define similarity or difference for graphs (see Discussion).

In the case of mitochondrial graphs, the most natural type of definition would be based on how hard it is to convert one graph to another, using the known mitochondrial dynamic transitions like fission or fusion. For the general case of mitochondrial graphs *MG*, determining such an "edit distance" is a challenging combinatorics problem. Once we simplify the space to the two-dimensional lattice of *mG*, it becomes possible to define a distance just based on the composition of morphisms, which we can do simply by counting the number of steps taken in the lattice representation of *mG* to get from one equivalence class to another. An important caveat is that because tip-to-side fusion does not have a direct inverse, these distance metrics are asymmetric; that is, the distance from A to B is not necessarily the same as the distance from B to A. The following is a definition of distance in *mG*, see Methods for derivation.

#### Proposition 2.5

Let $\left[ p, n\right] , \left[ p', n'\right] \in Ob(mG)$ be vector representations of mitochondria graphs. The asymmetric taxicab distance is

$$d_{\text {taxi}} \left( \begin{bmatrix} p \\ n \end{bmatrix}, \begin{bmatrix} p'\\ n' \end{bmatrix}\right) = {\left\{ \begin{array}{ll} \left| \frac{\Delta n + \Delta p }{2} \right| + \frac{\Delta n - \Delta p}{2}, & \Delta p \le \Delta n\\ \left| \Delta n \right| + \frac{\Delta p - \Delta n}{2}, & \Delta p \ge \Delta n \end{array}\right. } $$

where

$$\begin{aligned} \Delta n = n'-n \quad \text {and} \quad \Delta p = p'-p \end{aligned}$$

We note that interpreting the distance in terms of simply the number of morphological operations needed to get from one graph to another is an extreme simplification that ignores the relative rates of the different operations. As numerical data regarding the rates of fission. fusion, outgrowth, etc become available in a given system, these could be used to form a weighted distance function which would be an improvement over our current simplified metric. Implementing a more data-informed edit distance metric would also need to take into account the fact that there may be more ways to apply a given operation to one graph compared to another.

Unfortunately, we are currently unable to find a similar distance for the representation *mmG*. Actually finding the minimum is difficult because of the need to specify which connected components any given transformation will operate on. This can however lead to an algorithm that searches a constrained subset of all possible paths between two mitochondria graphs in order to determine the exact distance. Such an algorithm may have a time complexity that is small enough to be implemented on a computer. At the very least, we can obtain an upper and lower bound on the distance between any two mitochondria graphs that only requires us to know the multiset vector representation used in *mmG*. The following propositions provide lower and upper bounds on distance in *mmG*, with proofs given in Methods.

#### Proposition 2.6

Lower Bound: Let *M* and $M^{\prime }$ be mitochondria graphs where $n,n^{\prime }$ are the total number of degree-three vertices, $p,p^{\prime }$ are the total number of degree-one vertices, and $C,C^{\prime }$ are the number of connected components respectively. If $C^{\prime }\geq C$, then

$$|C'-C| + d_\text {taxi} \left( \begin{bmatrix} p +2|C'-C|)\\ n \end{bmatrix}, \begin{bmatrix} p'\\ n' \end{bmatrix}\right) \le dist(M,M') $$

Where dist(M,M’) is the minimum number of morphological operations required to convert from mitochondrial graph M to mitochondrial graph M’. If $C^{\prime }<C$, then

$$\begin{aligned} \frac{\Delta p - \Delta n}{2} + 2\,L + \max \{0, -\Delta n, \Delta n - L\} \le dist (M,M') \end{aligned}$$

where

$$\begin{aligned} L = \max \left\{ |C'-C|, \frac{\Delta n - \Delta p}{2}\right\} \end{aligned}$$

#### Proposition 2.7

Upper Bound: Let *M* and $M^{\prime }$ be mitochondria graphs where $n,n^{\prime }$ are the total number of degree-three vertices, $p,p^{\prime }$ are the total number of degree-one vertices, and $C,C^{\prime }$ are the number of connected components respectively. Then

$$\begin{aligned} dist(M,M') \le n' + \frac{3n-p}{2} + |C'-C| + C \end{aligned}$$

A method for comparing graphs has previously been described, known as Degree Distribution Quantification and Comparison (DDQC), which is based on comparing graphs based on the similarity of their degree distributions, using a manhattan type distance (Aliakbary et al. 2013). Our method is almost identical to that method subjected to the constraints of only having degree 1 or 3 vertices, except that the tip-to-side fusion operation will end up giving a shorter distance than the pure Manhattan distance.

One major limitation of this distance metric in its current form is that it assumes that the different morphological processes occur at comparable rates. Given more data about rate constants from biological images, it would be straightforward to modify the distance function to give more or less weight to specific operations, but such a modification would still be limited because it would assume that the rates are independent of the graph structure. It is likely that this may not be the case, and that for example a greater availability of tips might increase the rate of tip-side and especially tip-tip fusion. Previous modeling studies have incorporated just such assumptions (Sukhorukov et al. 2012; Zamponi et al. 2018), and simulated mitochondrial graph evolution using either differential equations to track the number of vertices of each time, or stochastic agent based simulations, with the notable finding that the steady-state distribution of graph structures depends only on the ratios between fission and fusion rates rather than on the rates themselves, allowing these ratios to be estimated from empirical image data. In order to align with that work, it would be necessary to modify the distance function by providing a weight for each transition that depends on the number of pendant versus internal edges, which in turn can be calculated from p and n.

Other factors that could influence the rates of transitions include, as noted above, the fact that in addition to a single large component, yeast mitochondria often include a number of small isolated segments. Tip-side fusion of these with the main component would effectively increase the rate of apparent branch outgrowth, such that the reservoir of small segments might act like a hidden variable. Our current framework would not be able to account for such effects. Another possibility is that the same physical migration of edges that occurs during vertex flip could allow on pendant edge to slide to the end of another creating one long pendant edge. This would represent a branch resorption operation but the mechanism would be entirely different and the rates would depend on the number of pendant edges. The effects of a tip number-dependent branch resorption process on network structure have not, to our knowledge, been modeled or studied.

### Biological Application 1: Analyzing the Distributions of Mitochondrial Graphs

Questions about mitochondrial morphology are inherently statistical. Given the huge space of possible mitographs, we should not expect the exact same graph to be seen over and over again. Instead, if we want to say anything about what the mitochondrial morphology is like in some particular cell type or set of growth conditions. we need a way to depict the distribution of graphs. The complexity of the set of possible mitographs MG makes this extremely difficult to do, and in particular it makes visualization of such a distribution virtually impossible. A major advantage of *mG* compared to either *MG* or *mmG* is that it can be easily depicted in a two dimensional lattice structure which makes it straightforward to visualize the distribution of mitochondrial graphs structures in actual data.

A second way that *mG* facilitates statistical analysis is that the [*p*, *n*] classes represent bins that represent multiple different graphs. This allows us to overcome the sparsity with which the full space of graphs would be covered by actual data, and have enough points in a bin to allow meaningful statistical testing. A third feature of our representation that helps support statistical analysis is the presence of a distance function. Many statistical tools already exist for statistical analysis of spatial data on a lattice, including methods for comparison of distributions and inference of underlying models (Cressie 1993). All of these statistical methods rely on two features: clustering datapoints into points on a lattice, which in this case is accomplished by the formation of the vertex flip equivalence classes, and the existence of a neighborhood structure that defines which lattice points are adjacent, which in this case is directly given by the morphisms that relate adjacent objects in *mG*.

**Fig. 14:**
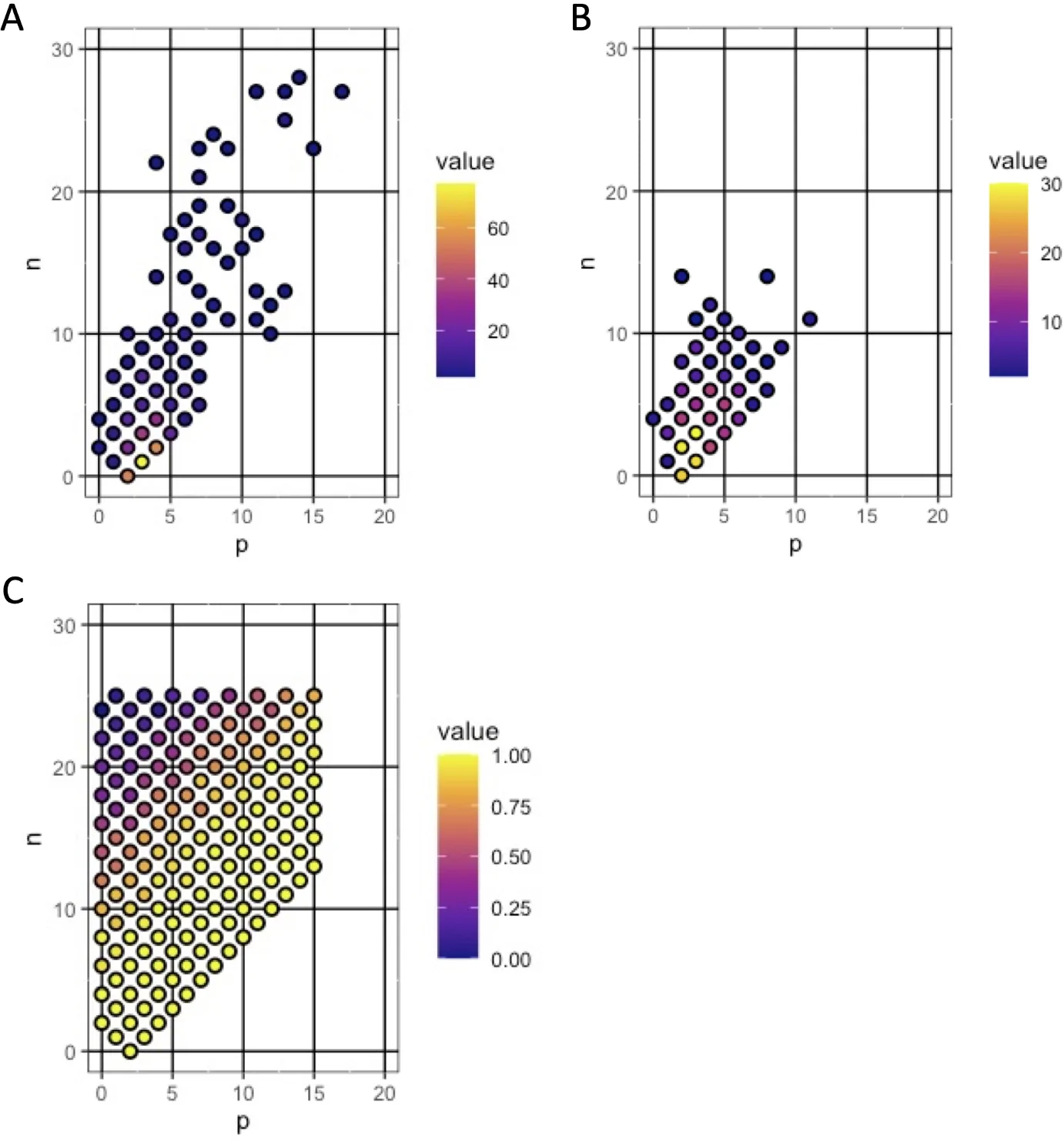
Using *mG* to visualize real data and model results. **A** Distribution of [*p*, *n*] for wildtype yeast data from Viana et al. (2020). Color indicates the number of mitographs for which the largest component fell into class [*p*, *n*] with colorbar on right indicating which colors correspond to which numbers. **B** Distribution of [*p*, *n*] for double-deletion yeast mutant data from Viana et al. (2020). **C** Plot showing the proportion of non-planar graphs in each [*p*, *n*] class for random graphs generated using the configuration model as described in the text. Yellow indicates cases where none of the random graphs were non-planar (Color figure online)

We illustrate this application by visualizing the distribution of mitochondrial graphs within *mG* in real data, using the wild-type budding yeast mitograph data published by Viana et al. (2020). As shown in Fig. 14A, we computed the number of degree 1 and 3 vertices from mitochondrial graph data reported in Viana et al. (2020) who used 3D imaging of budding yeast cells followed by application of MitoGraph software to generate graph representations of mitochondria in individual cells. Comparing the real data to the diagram of *mG* shows a broad distribution of vectors seen in real cells suggesting that much of the state space is accessed, but a clear concentration of graphs in the region around [2-4,0-4].

Although it is visually clear that the data is not uniformly distributed over the [*p*, *n*] bins, the binning of data on a 2D grid with a clear neighborhood structure does allow statistical tests for spatial randomness and correlations. To illustrate this idea, we apply Moran’s I test (Moran 1950) for spatial correlation using data from Fig. 14A, for n and p values up to 10, which incorporates almost all of the observations while avoiding the region of sparse coverage for larger graphs. The values of I can range from -1 to +1 with positive values reflecting positive correlation between neighboring locations. We obtain a value for Moran’s I of 0.400700032, indicating positive correlation, and p-value = 4.554e−15, strongly rejecting complete spatial randomness. We note that in order to take advantage of existing packages for calculating Moran’s I statistic, we used the $(n_b,n_a)$ labeling method based on the number of branch outgrowth and fusion operations described above, which directly maps the equivalence classes onto a simple 2D matrix of adjacent positions.

### Biological Application 2: Comparing Mutant and wt Mitograph Distributions

One of the reasons for thinking that mitochondrial network morphology is functionally relevant is the fact that the morphology varies between conditions, cell type, disease state, etc. Understanding such variation requires ways to measure and visualize differences in mitochondrial graph distributions between two different populations of cells. Likewise, genetic analysis hinges on being able to distinguish different mutant phenotypes. All of this requires a way to define what a difference is. one way is to use hand-crafted features. Our representation allows an alternative approach that avoids the need to guess features and just uses the structure of the space, based on the underlying assumption that a shift in class occupancy is more significant the farther away the shift is in terms of edit distance.

The published data reduce to raw counts of the two cell types (wt and mutant) for different graphs. Due to the vastness of the space of possible graphs, and the limited number of cells that can be analyzed in an experimental dataset, most graphs only occur once if at all, leading to a very sparse coverage of the space by the data, which makes both visualization and statistical comparison exceedingly difficult.

Instead, by combining all graphs with the same [*p*, *n*] into the same bin, we have the possibility of comparing distributions for the two cell types over those bins. One way would simply be to treat each bin independently and arrange them into a contingency table, allowing standard test to be applied. However, this approach throws away information about similarity between graphs. Ideally, we would consider two distributions more similar if they resulted in small shift in density on the [*p*, *n*] lattice.

Here we illustrate this approach by comparing the distribution of mitographs in wt yeast compared to yeast that are defective in both fission and fusion, again using data from Viana et al. (2020). Figure 14B depicts the distribution of [*p*, *n*] values seen for the double delete mutants, which can be visually compared to the distribution for wt cells in panel A.

As can be seen in both plots, and as confirmed by the results of Moran’s I test in the previous section, there is substantial correlation between occupancy in neighboring positions in the plot. Therefore, we cannot simply treat the bins independently in a contingency table test. Instead, order to compare the distributions statistically, we need a test that takes into account the neighborhood structure of the data. Here we use the Earth Mover Distance (EMD), a method that compares spatial distributions across 2D arrays by measuring the extent to which probability density has to be shifted between bins separated by different distances (Rubner et al. 1988). We again used the $(n_b,n_a)$ labeling method to map each dataset onto a simple 2D matrix, thereby allowing both datasets to be used as input in existing software for computing EMD (see Methods). From the two datasets describing WT and double delete mutant mitographs, we obtain an earth mover distance of 0.764836, which means that the two distributions can be brought into alignment by shifting probability density on average less than 1 bin away. A permutation test on the data indicates that this result is significant with P<0.001 showing that the two datasets can be clearly distinguished based on this test.

This result emphasizes how the reduced representation allows us to compare the distribution of mitographs between two different strains. This is a difficult problem if we just consider the entire space of possible graphs, but by using the simplified representation we provide a way to effectively "bin" the mitographs, allowing numbers in different bins to be compared.

### Biological Application 3: Comparing the Distribution of Planar Versus Non-planar Random Mitograph Models

In some organisms, including budding yeast, the mitochondria are physically anchored on the cell cortex (Lackner et al. 2013; Kraft et al. 2017). Consequently, when mitochondrial graphs are analyzed in budding yeast, they are seen to be planar (Viana et al. 2020). Interestingly, when mitochondria are compared between wild type and the double deletion mutants, it was found the planarity constraint was eliminated in the latter (Viana et al. 2020). We therefore hypothesized that perhaps this loss of the planarity constraint might lead to a re-distribution of graphs among the possible equivalence classes.

We can use the [*p*, *n*] representation plot to get a visual insight into the possible effect of removing a planarity constraint. To do this, we generated, for each [*p*, *n*], a set of random graphs using the configuration model of random graphs (see Methods), taking only those that retain a single connected component, and then asked what fraction of those randomly generated graphs are planar. The result is plotted in Fig. 14C. We can understand this plot qualitatively by first considering the left edge where p=0. The graphs on this edge consist entirely of regular cubic graphs. All regular cubic graphs are planar below order 6, at which point the first non-planar regular cubic graph $K_{3,3}$ occurs, but after that, the fraction that are planar rapidly drops to zero as the size of the graphs increases. Moving away from the left edge, we see the fraction of planar graphs goes up as p increases due to branch fission ($a^{-1}$), which we can understand because as p increases with n fixed, each pendant edge that is added removes one degree 3 vertex from the cubic core, instead using it as the base of the pendant edge. If instead we consider the operation of branch outgrowth, which increases both n and p by one, represented by diagonal movements up and to the right on the plot, in this case each time a new branch grows out, the cubic core is preserved, and thus the fraction of planar graphs stays the same, due to the fact that pendant edges do not affect planarity.

The same arguments that explain the appearance of Fig. 14C, also show that the loss of the planarity constraint in the double deletion mutant is unlikely to explain the difference in the occupancy of the [*p*, *n*] classes in the double delete versus wild type strains. When we compare the wild type and double delete mutants, the biggest difference between the two strains occurs for values of n less than 6. Since there are no non-planar graphs with n less than 6, the planarity constraint cannot affect the distribution of graph counts among the equivalence classes in that region of the diagram.

## Discussion

### Comparison with Other Measures of Graph Similarity

Here we use the morphological operations of mitochondrial dynamics to define a distance function and neighborhood structure that allows us to bin, visualize, and perform statistical analysis on sets of mitochondrial graph structures derived from microscopic images. The essence of our approach is to view graphs as being similar or different based on how many sequential morphological operations would have to occur inside the cell to convert one graph to another. We believe that this approach provides a natural way to simplify the space of mitographs. The fact that mitochondrial dynamic processes like fission and fusion may themselves contribute substantially to transport of material by mitochondria (Holt et al. 2026), is another compelling reason to use mitochondrial dynamics, rather than arbitrary graph features, to define the space of mitographs.

This approach is quite different from many alternative definitions of graph similar that are based on direct comparison of structural similarity (Haber and Schneidman 2022). The fact that our approach when applied to real data gives high correlation between neighboring classes and can resolve differences between wt and mutant strains suggests that the similarity metric based on the limited set of operations we have considered is in fact able to capture aspects of graph similarity of actual mitochondria. This might not be the case if, for example, there were additional morphological operations having strong effects different from the ones we consider, or if selection was acting on mitochondrial graph features not captured by our current analysis of those operations. For example, in the graphlet or substructure kernel methods for comparing graphs, distance depends on the presence of specific sub-graphs such as loops of certain sizes. Our metric does not distinguish such features, such that graphs contained within a similar [*p*, *n*] might differ in their graphlet composition and therefore be given a non-zero distance. If wt mitochondria had a strong tendency to favor such a feature, it would be spread across many different equivalence classes in *mG* such that we might not see the clear peak in the data distribution.

A conceptually simple method for comparing graphs would be to measure some number of graph descriptors, record each graph as a point in this many-dimensional feature space, and then define a distance between point clouds that represent clusters of similar graphs. Depending on which features are chosen, this approach may or may not produce a similar binning of graphs as our current method. In any case the big difference is that the feature-based method relies on human intuition about what features to measure, while the current algebra based method only relies on knowledge of the dynamic processes that interconvert mitochondrial graphs, and then the bin structure is induced by the structure of those operations.

One measure of graph similarity that is similar in spirit to our distance in using notions of dynamically interconverting graphs, is the Graph Edit Distance (GED; Sanfeliu and Fu 1983). This measures the minimum number of edges or vertices that need to be added, deleted, or substituted in order to convert one graph into the other. This is not equivalent to our measure, and it is easy to come up with pairs of graphs that are vertex flip equivalent but which have a non-zero GED. More importantly, GED involves operations that are not biologically plausible.

Another measures of graph similarity related to the distance that we define is a test that uses the Kolmogorov-Smirnov statistic to test similarity of degree distributions (Tomlinson et al. 2022). This relates directly to our distance which is defined in terms of degree sequence, but it is not actually a way to compare two individual graphs, rather a way to compare distributions of graphs. We previously noted the Degree Distribution Quantification and Comparison (DDQC) method, which is based on comparing graphs based on the similarity of their degree distributions, using a manhattan type distance (Aliakbary et al. 2013), which is almost the same as our distance except that it does not take into account tip-side fusion, which we feel is important given its prevalence compared to tip-tip fusion.

### Algebraic Structure of *mG*

As currently defined, the category *mG* has some morphisms for which there is no inverse, and has some objects that are not the source of some morphisms because the morphological operations represented by those morphisms would produce graphs that do not belong to MG. Given that the morphisms are composable, and every object has an inverse which is the null transformation, *mG* constitutes a category, but this appears to be the most that we can say about its algebraic structure.

In formulating our morphological operations, we have stated that tip-side fusion does not have an inverse. This may not be strictly correct given the challenge of detecting such an event compared to fission in the middle of a branch that is usually very obvious. The technical challenge is that when one sees an apparent fission event, how does one decide if it is really fission of a previously connected structure, versus a moving apart of two non-connected features that happened to overlap enough that they appeared to be in contact given the limited resolution of light microscopy. For fission in the middle of a branch, it is usually quite obvious that the branch was previously connected because it coheres for a long time as it moves back and forth in the cell. The odds that such an apparently connected branch are actually two unconnected branches whose tips happened to stay near each other as they both move around randomly in the cell, is very low. On the other hand, it is easier to imagine two branches that happened to cross each other without being fused together, such that if one sees an apparent fission event in which a one branch detaches at its base, it would be easy to discount. Really showing that tip-side fusion occurs would require higher spatial and temporal resolution, and will hopefully be decided one way or another by current advanced methods (Stepp et al. 2026).

If it turns out that tip-side fusion really does have an inverse, then *mG* would be a category in which every morphism is an isomorphism, which is the definition of a groupoid. It would still not be a group because of the boundary conditions, which represent objects for which certain morphisms cannot be applied.

### Biological Ramifications of Tip-Side Fusion

In addition to affecting the algebraic structure of our representation, the question of whether or not tip-side fusion has an inverse may also affect biologically relevant aspects of mitochondrial structure. The graph from Fig. 9 suggests that, in a random walk on the state-space with all transitions chosen at equal probabilities, the asymmetry created by the presence of tip-to-side fusion could produces a drift towards mitochondria graphs that have a high ratio of degree-three vertices relative to degree-one vertices. However, we note that any predictions about the impact of unbalanced tip-side fusion will ultimately depend on the relative rates of the different processes. If fission and branch resorption occur at a sufficiently high rate, it would counter-act any bias created by tip-side fusion. Such an effect is likely to be the case given our analysis of experimental data in Fig. 14A, in which the distribution shows no sign of being focused near the left boundary of the plot. On the contrary, the fact that most observed mitographs tend to lie near the opposite extreme, it might be the case that tip-side fusion exists to resist a bias created by high rates of fission. As implied by the findings of Gatti et al. (2023), the role of tip-side fusion could be tested experimentally by inhibiting actin-mediated processes and looking for any alteration in the mitograph distribution.

### Representing Mitochondrial Growth and Division

We have only considered biological processes that change the mitochondrial graph structure locally by acting on individual vertices and edges. But there are two major morphological transitions that we currently ignore, namely, the increase of mitochondrial network size by addition of new material, and the dividing up of the mitochondrial network during cell division.

The partitioning between the mother and daughter cells in the case of yeast will impose a finite constraint on the size of the graphs in *MG* or the range of p and n values in the simplified [*p*, *n*] representation. It is not obvious how to allocate different components to different cells. At a mechanistic level, partitioning mitochondria into daughter cells may involve different fission operations than those that occur when a cell is not dividing. We have already defined a disconnected fission operation that acts on a cut-edge. it may be necessary to define a special divisional fission operation that can separate components lacking a single cut-edge. An alternative suggested by live images of mitochondrial partitioning into the yeast bud is that it may represent a form of branch outgrowth, with a single long branch extending into the bud, and then establishing its own network morphology at a later stage. Within our representation framework, we hypothesize that the newly formed bud might start out with a long single I-tubule [2, 0] and then gradually execute some form of random walk over *mG* as the mitochondrion grows and adds additional branches.

One potentially tractable question is whether mitochondria graphs in dividing cells have any discernible differences from mitochondria graphs in a non-dividing cells. Structural changes in mitochondria have been linked to changes in the cell cycle (Daciana et al. 2002). A particularly interesting experiment would be to image how a mitochondrial graph is split during cell division, in order to determine whether there any definable rules that specify or predict the cut set for the edges by which a connected component is split between the sister cells. One possibility is that division might entail an imbalance between fission and fusion so as to produce more components, but another is that some entirely separate machinery may be involved in pulling the network into two pieces.

With respect to representation of mitochondrial structure as a function of network size, the key question is whether biological processes are affected more by global network structure, or by local "intensive" properties of the network. As cells grow, the content of mitochondria grows proportionally (Rafelski et al. 2012; Chacko et al. 2025). Since the density of mitochondria stays constant one could imagine that its intensive properties are maintained. On the other hand, as cell size increases, changes to network organization may be required to allow transport to scale with increased cell volume. Current evidence suggests that both global and local properties may be important (Viana et al. 2020). Our current representation is strictly global - expanding a network by adding more branches with the same type of organization, for example by pasting together two similar networks, would produce a network that falls in entirely different region of *mG*.

### Defining Cell States Based on Mitochondrial Morphology

There has been growing interest in enumerating cell types and cell states based on cluster analysis of large datasets, for example of gene expression data. The basis of such approaches is clustering - having a way to determine the distance between two cells in some kind of a state space and then clustering cells based on proximity in that space. Our algebraic representation of mitochondrial dynamics provides a way to do this, because we can quantify a “distance" based on the minimum number of operations required to convert one equivalence class to another. The approach would be to collect mitochondrial graph structures for a large number of cells, and then calculate distances between pairs of cells based on the distance calculated from the [*p*, *n*] representation. One potential advantage of this approach compared to other ways to define cell state based on clustering, is that we already know a lower and upper bound on the distance, as shown in Propositions 2.6 and 2.7. Such a distance-based clustering would then lead to two general types of applications. First, the clusters would define different cell states in terms of their mitochondrial network structure. Second, the effects of genetic or metabolic perturbations could be mapped and visualized either onto the [*p*, *n*] representation itself as in Fig. 14 A,B, or onto the clusters that result, either way allowing different phenotypes to be distinguished from each other in new ways.

The key to this approach is the great simplification that results in going from MG to *mG* based on vertex flip equivalence. We will close by discussing whether this approach could be generalized to other aspects of biological structure.

### An Algebraic Approach to Cell Representation

It has long been a goal of cell biology to define cell state in some rigorous, quantifiable way, that would allow different cell types to be distinguished and related to their molecular function. One approach has been to use transcriptomics to classify cells based on gene expression patterns. However, we recognize that cell state at a purely molecular level will likely involve not only gene expression but also protein quantity, post-translational modifications, metabolites, etc. Simultaneously measuring all of these molecular scale variables in individual cells is currently a huge technological challenge, and most studies have only measured one type of variable such as transcriptomics or proteomics. Moreover, these "omics" methods are generally destructive measurements and cannot be used to measure how the system changes over time.

As an alternative strategy, it has been recognized that much of cellular function is reflected in organelle-scale cellular organization. High throughput automated microscopy, combined with increasingly powerful methods for image analysis and segmentation, have resulted in the ability to extract large amounts of quantitative data from microscopy images of cells at large scale (Bagheri et al. 2022; Scipioni et al. 2024; Seal et al. 2025). There has thus been growing interest in recent years in using such microscopy image data, including from live cells, to develop ways to cope with the immense complexity of molecular detail in cells, by representing cellular structure and cell state using coarser-grained representations, such as at the level of organelle morphology (Johnson et al. 2015; Murphy 2016; Rafelski and Theriot 2024). The tools currently being used to build such representations are largely drawn from statistics, using principal components analysis (PCA) and other dimensionality reduction methods to take a large number of morphological descriptors (sometimes referred to as the "morpholome"), ranging from feature descriptors like sphericity produced by standard image analysis packages to more advanced feature descriptors such as terms in a spherical harmonic expansion representing an organelle surface. By combining such measurements into a small number of modes via PCA or other dimensionality reduction methods, it is possible to produce a low dimensionality representation that can be visually comprehended and within which dynamics can be modeled in terms of a vector field (Chang and Marshall 2019). Another approach that is currently seeing increased use is to employ machine learning methods such as autoencoders or GANs to learn how to encode the structure in a collection of images (Chan et al. 2020; Sun et al. 2022; Donovan-Maiye et al. 2022; Wu et al. 2022). This approach avoids the need to extract image features, but at the expense of interpretability compared to PCA based methods. Both of these methods have, in common, the fact that they use the images themselves, either directly or via extracted features, as the basis for defining a state space to represent cell structure.

Here we have described an alternative approach to constructing a cell representation state space. In contrast to the PCA-based morpholomic analysis based on image features or machine learning on image regions, both of which ultimately use structure as the basic element, our approach focuses on morphological processes that convert the shape of an organelle into a different shape. These operations form an algebraic structure that can be used to represent the possible states of a cell in terms of transitions between the states, and that then allows tools from abstract algebra to be brought to bear.

The challenge is that the state space, such as the space of all possible graphs, can already be very complex, and so, as with the morphological features-based methods, we need a way to reduce the complexity of the space. The approach we advocate here is to select one of the morphological processes, in our case vertex flip, and use it to construct an equivalence relation. Given this relation, we can then define a new state space in which the elements of the space are not individual shapes (for example mitochondrial graphs) but rather equivalence classes defined by the relation. This results in a vast simplification of the state space. This same approach can, in principle, be applied to any cellular structures for which an equivalence relation can be defined in terms of some set of morphological operations. By basing the reduction in space complexity on the underlying dynamics of the cellular processes, this method provides a more direct link to underlying mechanisms than would be possible with a purely statistical analysis of image features.

## Summary

In conclusion, applying abstract algebra to the study of mitochondrial dynamics not only provides a formal way to represent structure as a framework for modeling or for quantifying similar states, it deepens our understanding of cellular structures and inspires experimental questions that might otherwise have remained unexplored. By conceptualizing the morphological transformation of mitochondria as generators of an algebraic structure, and identifying a transformation that can define an equivalence relation, we are prompted to consider finite constraints, cyclic motifs, and the algebraic properties of mitochondrial networks in ways that traditional biological approaches might overlook. This theoretical framework thereby leads to new experimental designs that would not have emerged from a purely empirical approach.

## Methods

### Visualizing Mitograph Data

In order to plot actual data onto the [*p*, *n*] representation, we used a custom r script that reads the.gnet files produced by the mitograph software, and then uses the igraph package to convert the.gnet file into a graph using the graph_from_edgelist() function,, extract the largest component using the decompose() function, and then extract p and n using the degree_distribution() function. Any graph that is not a valid mitograph, assessed based on the presence of vertices with degree other than 1 or 3, is discarded. The remaining data (n=595 for WT, and n= 362 for double delete mutants) are used to compute the number of graphs that fall into each of the [*p*, *n*] classes. Comparison of distributions using earthmover distance (EMD) was carried out using the emd2d function in r with a permutation test to obtain a p-value. Testing the fraction of planar random graphs was carried out using a configuration model using igraph and the Boyer Myrvold Planarity Test from the RBGL package. Scripts are available on github at https://github.com/WallaceMarshallUCSF/mitograph_data_analysis.git.

### Derivation of (r,E) Labeling in Terms of [p,n] Labeling

From the definition of cyclomatic number for graphs of one component

$$\begin{aligned} p + n = E - r + 1 \end{aligned}$$

and from the Handshaking Lemma, we have

$$\begin{aligned} p + 3n = 2E \end{aligned}$$

which gives the number of edges:

$$\begin{aligned} E = \frac{3n+p}{2} \end{aligned}$$

The number of vertices if just V=p+n.

Combining the number of edges and vertices, we get the cyclomatic number

$$\begin{aligned} r = \frac{n-p}{2} + 1 \end{aligned}$$

with the inverse relations

$$\begin{aligned} p = \frac{E-3r+3}{2} \end{aligned}$$

$$\begin{aligned} n = \frac{r+E-1}{2} \end{aligned}$$

### Derivation of Lowest Bound of E Versus r in (r,E) Labeling

Since p≥0 we get from the equation for p above

$$\begin{aligned} \frac{E-3r+3}{2} \ge 0 \end{aligned}$$

hence

$$\begin{aligned} E \ge 3r-3 \end{aligned}$$

However, E cannot be negative, leading to several special cases when r is less than 2.

Case r=0, To make *p* an integer, *E* must be odd (since E-3(0)+3=E+3 must be even). The smallest positive odd integer is E=1. This yields p=2 and n=0 (a single edge connecting two degree-1 vertices, $K_2$). Therefore, in this case

$$\begin{aligned} E_{min} = 1 \end{aligned}$$

Case r=1 Since p is an integer, E-3(1)+3=E must be even. E can’t be zero or else we would have an empty graph. The next smallest even integer is 2. inserting this value into the equations for p and n yields p=1 and n=1, i.e. a vertex with a self-loop and a single pendant edge.

Case r≥2 For any r≥2, the boundary condition E=3r-3 yields p=0 and n=2r-2, both integers, describing a regular cubic multigraph.

For r=2, $E_{min} = 3$, describing two vertices sharing 3 parallel edges.

For r=3, $E_{min} = 6$, describing the complete graph $K_4$.

### Derivation of Single Integer Class Label

Considering the diagram in Fig. 9, the left boundary is given by

$$\begin{aligned} p+n \ge 2 \end{aligned}$$

and the right boundary by

$$\begin{aligned} p \le n+2 \end{aligned}$$

so the number of objects (equivalence classes) in any given row defined by a given value of n, is

$$\begin{aligned} \lfloor \frac{n}{2} \rfloor +2 \end{aligned}$$

because the number of objects in a row increases every two rows. next we sum all the objects in the rows up to n-1:

$$\begin{aligned} objects \ before \ row \ n = 1+ \sum _{k=1}^{n-1}{(2+\lfloor \frac{k}{2} \rfloor )} \end{aligned}$$

which can be rewritten

$$\begin{aligned} objects \ before \ row \ n = 1+ (2n-1)+ \sum _{k=1}^{n-1}{(\lfloor \frac{k}{2} \rfloor )} \end{aligned}$$

The final summation can be expressed as the product of two consecutive halves of n to yield

$$\begin{aligned} objects \ before \ row \ n = 1+ (2n-1)+ \lfloor \frac{n-1}{2} \rfloor \lfloor \frac{n}{2} \rfloor \end{aligned}$$

Finally the position of a given object in a given row is

$$\begin{aligned} position \ in \ current \ row = 1 + \lfloor \frac{p}{2} \rfloor \end{aligned}$$

Adding the position in the current row to the number of objects in all the rows below, and then simplifying, we obtain a unique object label as a function of p and n:

$$\begin{aligned} label = 2n + \lfloor \frac{n-1}{2} \rfloor \lfloor \frac{n}{2} \rfloor + \lfloor \frac{p}{2}\rfloor \end{aligned}$$

### Derivation of Asymmetric Taxicab Distance

#### Proof

There exists $\alpha ,\beta \in \mathbb {Z}$ and $\gamma \in \mathbb {N}$ such that

$$\begin{bmatrix} p \\ n \end{bmatrix} + \alpha \begin{bmatrix} 2 \\ 0\end{bmatrix} + \beta \begin{bmatrix} 1\\ 1\end{bmatrix} + \gamma \begin{bmatrix} -1 \\ 1 \end{bmatrix} = \begin{bmatrix} p' \\ n' \end{bmatrix} $$

the taxicab distance is

$$\begin{aligned} \min _{\gamma \ge 0} \left\{ |\alpha | + |\beta | + \gamma \right\} \end{aligned}$$

constrained by the above linear equation. We thus solve the minimization problem.

$$\begin{aligned} \begin{bmatrix} 2 & 1 & -1\\ 0 & 1 & 1 \end{bmatrix}\begin{bmatrix} \alpha \\ \beta \\ \gamma \end{bmatrix}&= \begin{bmatrix} \Delta p \\ \Delta n \end{bmatrix} \\ \beta&= \Delta n - \gamma \\ \alpha&= \gamma + \frac{\Delta p - \Delta n }{2} \end{aligned}$$

and so

$$d_{\text {taxi}} \left( \begin{bmatrix} p \\ n \end{bmatrix}, \begin{bmatrix} p'\\ n' \end{bmatrix}\right) = \min _{\gamma \ge 0} \left\{ \left| \gamma + \frac{\Delta p - \Delta n }{2}\right| + \left| \Delta n - \gamma \right| + \gamma \right\} $$

Since this is a piecewise linear convex function, the minimum must occur either at the boundary γ=0, or, at a place where one of the absolute value terms is 0 – either when $\gamma = \Delta n$, which is feasible when $\Delta n \ge 0$, or $\gamma = \frac{\Delta n - \Delta p }{2}$, which is feasible when $\Delta p \le \Delta n$. After checking each of the possibilities, we arrive at the asymmetric taxicab distance defined in Proposition 2.5□

### Derivation of Lower Bound on Asymmetric Taxicab Distance

#### Proof

- If $C^{\prime }\geq C$, then the only possible way to produce the necessary additional connected components is through $|C^{\prime }-C|$ additional fission steps. Thus, we first perform $|C^{\prime }-C|$ fission steps, and then compute the taxicab distance from that point to obtain the lower bound.
- If $C^{\prime }<C$, then the only way to get rid of the excess connected components is through $|C^{\prime }-C|$ additional tip-to-tip and/or tip-to-side fusion steps (in this instance, mitophagy can be counted the same as tip-to-tip fusion on an I-tubule). Thus, similar to our proof of Proposition 2.5, we minimize
  $$\begin{aligned} \min \left\{ \alpha + |\beta | + \gamma + \epsilon \right\} \end{aligned}$$
  subject to the constraints
  $$\begin{bmatrix} 2 & 1 & -1 & - 2 \\ 0 & 1 & 1 & 0 \end{bmatrix} \begin{bmatrix} \alpha \\ \beta \\ \gamma \\ \epsilon \end{bmatrix} = \begin{bmatrix} \Delta p \\ \Delta n \end{bmatrix}, \quad \alpha ,\gamma ,\epsilon \ge 0, \quad \gamma + \epsilon \ge |C'-C| $$
  solving the linear equation constraint for α and β we get
  $$\begin{aligned} \beta&= \Delta n - \gamma \\ \alpha&= (\gamma + \epsilon ) + \frac{\Delta p - \Delta n}{2} \ge 0 \end{aligned}$$
  Hence,
  $$\begin{aligned} \gamma + \epsilon \ge L:= \max \left\{ |C'-C|, \frac{\Delta n - \Delta p}{2} \right\} \end{aligned}$$
  and our objective function becomes
  $$\begin{aligned} \min \left\{ \frac{\Delta p - \Delta n}{2} + 2(\gamma + \epsilon ) + |\Delta n - \gamma |\right\} \ge \frac{\Delta p - \Delta n}{2} + 2\,L + \min \left\{ |\Delta n - \gamma |\right\} \end{aligned}$$

Let $s = \gamma + \epsilon$, then, for fixed values of *s*, $|\Delta n - \gamma |$ is minimized by taking γ to be the integer in $\left[ 0, s\right]$ closest to Δn,

$$\min _{\gamma \ge 0} \left\{ |\Delta n - \gamma |\right\} = {\left\{ \begin{array}{ll} -\Delta n & \Delta n < 0 \\ 0 & 0\le \Delta n \le s \\ \Delta n - s & \Delta n > s \end{array}\right. }$$

Minimizing $s\in \mathbb {N}$ over s≥L, we arrive at the lower bound in Proposition 2.6. □

### Derivation of Upper Bound on Asymmetric Taxicab Distance

#### Proof

To obtain the upper bound, we find an upper bound on the distance from *M* to an intermediate graph, $H^{\prime }$, which is composed of $C^{\prime }$ I-tubules, and then find the exact distance from $H^{\prime }$ to $M^{\prime }$. By the triangle inequality, $dist\left(M,M^{\prime }\right)\leq dist\left(M,H^{\prime }\right)+dist\left(H^{\prime },M\right)$.

1. Distance from M to $H^{\prime }$ : Let $$\left[ p_i, n_i\right] $$ be a connected component in *M*. We first find $$d_{\text {taxi}} \left( [ p_i, n_i], [2, 0]\right) $$. Notice from Proposition 2.1 that
  $$\begin{aligned} 2 - p_i \ge - n_i\implies \Delta p \ge \Delta n \end{aligned}$$
  and so, since no matter where on any of the graphs represented by $\left[ p_i, n_i\right]$ we perform the transitions to get to the graph represented by $\left[ 2, 0\right]$, we will end up at an I-tubule, then
  $$d_{\text {taxi}} \left( \begin{bmatrix} p_i \\ n_i \end{bmatrix}, \begin{bmatrix} 2\\ 0 \end{bmatrix}\right) = |\Delta n | + \frac{\Delta p + \Delta n}{2} = n_i + \frac{ 2 - p_i + n_i}{2} = 1+\frac{3n_i}{2} - \frac{p_i}{2}$$
  is the exact edit distance between $$\left[ p_i, n_i\right] $$ and $$\left[ 2, 0\right] $$. If $C^{\prime }\geq C$, then the only way to produce the additional connected components is through $|C^{\prime }-C|$ additional fission steps. If we first transform *M* into the graph with *C* I-tubules, then perform the extra fission steps, we will end up with $C^{\prime }$ I-tubules. Similarly, if $C<C^{\prime }$, then we must perform at least $|C^{\prime }-C|$ additional fusion or mitophagy steps to end up with $C^{\prime }$ connected components. Since performing tip-to-side fusion on two I-tubules makes one I-tubule, we can follow the same process as before to get
  $$\begin{aligned} dist(M,H') \le \sum _{i=1}^C\left( 1 + \frac{3n_i}{2} - \frac{p_i}{2}\right) + |C'-C| = C + \frac{3n-p}{2} + |C'-C| \end{aligned}$$.
2. Distance from $H^{\prime }$ to $M^{\prime }$ : Let $$\left[ p_j, n_j\right] $$ be a connected component in $M^{\prime }$. We first find $$d_{\text {taxi}} \left( [2, 0], [ p_j, n_j] \right) $$. By Proposition 2.1, we have
  $$\begin{aligned} p_j-2\le n_j \implies \Delta p \le \Delta n \end{aligned}$$
  and so
  $$d_{\text {taxi}} \left( \begin{bmatrix} 2 \\ 0 \end{bmatrix}, \begin{bmatrix} p_j\\ n_j \end{bmatrix}\right) = \left| \frac{\Delta n + \Delta p }{2} \right| + \frac{\Delta n - \Delta p}{2} = \left| \frac{n_j + p_j-2 }{2} \right| + \frac{n_j - p_j+2}{2} = n_j$$
  is the exact edit distance. Hence,
  $$\begin{aligned} dist(H',M') = \sum _{j=1}^{C'}n_j = n' \end{aligned}$$.

□
